# High-Intensity Focused Ultrasound Increases Collagen and Elastin Fiber Synthesis by Modulating Caveolin-1 in Aging Skin

**DOI:** 10.3390/cells12182275

**Published:** 2023-09-14

**Authors:** Seyeon Oh, Do-Young Rhee, Sosorburam Batsukh, Kuk Hui Son, Kyunghee Byun

**Affiliations:** 1 Functional Cellular Networks Laboratory, Lee Gil Ya Cancer and Diabetes Institute, Gachon University of Medicine, Incheon 21999, Republic of Korea; 2 Leaders Clinic, Seoul 05065, Republic of Korea; 3 Department of Anatomy & Cell Biology, College of Medicine, Gachon University, Incheon 21936, Republic of Korea; 4 Department of Thoracic and Cardiovascular Surgery, Gachon University Gil Medical Center, Gachon University, Incheon 21565, Republic of Korea; 5 Department of Health Sciences and Technology, Gachon Advanced Institute for Health & Sciences and Technology (GAIHST), Gachon University, Incheon 21999, Republic of Korea

**Keywords:** aging, caveolin-1, collagen, high-intensity focused ultrasound, p53 activity, skin rejuvenation

## Abstract

Caveolin-1 (Cav-1) induces cellular senescence by reducing extracellular signal-regulated kinase (ERK)1/2 phosphorylation and activating p53 via inhibition of mouse double minute 2 homolog (MDM2) and sirtuin 1 (Sirt1), promoting cell cycle arrest and decreasing fibroblast proliferation and collagen synthesis. High-intensity focused ultrasound (HIFU) treatment increases collagen synthesis, rejuvenating skin. Using H_2_O_2_-induced senescent fibroblasts and the skin of 12-month-old mice, we tested the hypothesis that HIFU increases collagen production through Cav-1 modulation. HIFU was administered at 0.3, 0.5, or 0.7 J in the LINEAR and DOT modes. In both models, HIFU administration decreased Cav-1 levels, increased ERK1/2 phosphorylation, and decreased the binding of Cav-1 with both MDM2 and Sirt1. HIFU administration decreased p53 activation (acetylated p53) and p21 levels and increased cyclin D1, cyclin-dependent kinase 2, and proliferating cell nuclear antigen levels in both models. HIFU treatment increased collagen and elastin expression, collagen fiber accumulation, and elastin fiber density in aging skin, with 0.5 J in LINEAR mode resulting in the most prominent effects. HIFU treatment increased collagen synthesis to levels similar to those in Cav-1-silenced senescent fibroblasts. Our results suggest that HIFU administration increases dermal collagen and elastin fibers in aging skin via Cav-1 modulation and reduced p53 activity.

## 1. Introduction

Caveolae are 50–100 nm, flask-shaped invaginations in the plasma membrane that are enriched in proteins known as caveolins [1]. Two members of the caveolin family, caveolin (Cav)-1 and Cav-2, are highly expressed in fibroblasts, endothelial cells, adipocytes, and pneumocytes, whereas Cav-3 is primarily expressed in muscle cells [2]. Cav-1 contains a caveolin-scaffolding domain, which mediates binding with caveolin-binding motifs in regulatory proteins, such as protein kinase A, protein kinase C, endothelial nitric oxide synthase, and epidermal growth factor (EGF) receptor [3]. Cav-1 binding generally inhibits downstream signaling pathways [3], including those induced by growth factors or involved in cell cycle progression, ultimately decreasing cell proliferation [4,5,6]. Studies indicate that Cav-1 plays a key role in cellular senescence [7,8]. Senescent dermal fibroblasts exhibit increased expression of Cav-1 and several senescence markers, including p21, p16, and p53, accompanied by a decreased capacity for collagen synthesis [9,10,11]. Moreover, in aging skin from mice and humans, reduced collagen type I levels are accompanied by Cav-1 upregulation [12]. In addition to effects on collagen synthesis, increased Cav-1 expression in senescent dermal fibroblasts is associated with reduced extracellular signal-regulated kinase (ERK)1/2 phosphorylation [13]. During cell growth, EGF stimulates ERK1/2 phosphorylation [13], and the Cav-1-mediated loss of ERK1/2 phosphorylation decreases cell proliferative capacity [14]. ERK1/2 phosphorylation upregulates cyclin D, resulting in cell cycle progression from G0/G1 to S phase [15,16,17,18]. Cyclin-dependent kinase 2 (CDK2) also increases S-phase entry by forming a complex with cyclin E [19,20]. Cav-1 overexpression in young dermal fibroblasts leads to decreased ERK1/2 phosphorylation and premature cellular senescence [13]. However, inhibition of Cav-1 with small-interfering (si)RNA restores EGF-mediated ERK1/2 activation [7].

Cav-1 also contributes to cellular senescence through the modulation of p53, a primary inducer of senescence [9]. Cav-1 overexpression upregulates both p53 and p21, which together promote cell cycle arrest, leading to cellular senescence [5,9,21]. The nuclear localization of mouse double minute 2 homolog (MDM2), an E3-ubiquitin ligase, promotes p53 degradation; however, Cav-1 binding to MDM2 results in the cytoplasmic sequestration of MDM2, increasing p53 activity [9,22]. Similarly, Cav-1 promotes p53 activation by inhibiting sirtuin 1 (Sirt1), which mediates p53 deacetylation and inhibition [23].

Extracellular matrix (ECM) destruction, together with reduced collagen fiber accumulation, leads to reduced skin elasticity and promotes skin wrinkling [24,25]. During aging, matrix metalloproteinase (MMP) levels increase, enhancing the MMP-mediated degradation of the ECM [26]. Tissue inhibitors of metalloproteinases (TIMPS) are endogenous inhibitors of MMPs [27]. Collagen, one of the significant elements of ECM, is mainly secreted from fibroblasts, and balance between MMPs and TIMPs is essential to maintain ECM structure [28,29]. Notably, Cav-1 may contribute to this process by inducing MMP2 and MMP9 expression [30].

Hyperthermic injury can trigger collagen synthesis and induce a skin-lifting effect [31], leading to the development of various skin rejuvenation modalities that trigger collagen remodeling by subjecting a targeted area of the skin to controlled thermal injury, including laser resurfacing, radiofrequency (RF) therapy, microfocused ultrasound, and high-intensity focused ultrasound (HIFU) [32,33,34,35,36].

The application of acoustic energy produced by HIFU to the skin can raise local temperatures to approximately 60–70 °C, promoting skin rejuvenation and tightening by inducing collagen denaturation, remodeling, and synthesis [37,38,39,40,41]. Because HIFU generates acoustic energy, deeper tissues can be reached by HIFU than can be reached by laser or RF therapies [42,43,44]. The penetration of ultrasound waves into skin tissues induces the vibration of molecules in the region in which the ultrasound beam is focused, resulting in friction that generates heat and thermal injury [42,43,44].

During HIFU treatment, the penetration depth is determined by the wave frequency, with higher frequencies resulting in shallower focal injuries and lower frequencies able to penetrate more effectively to induce thermal injury in deeper tissues [45]. In parallel, the rate of heat generation in tissues is determined by both frequency and intensity, with higher frequencies and intensities leading to more rapid heat generation in the focal area [46,47,48]. At high intensities, HIFU induces rapid cell death via coagulative necrosis [47,48]. Therefore, HIFU has also been leveraged for decreasing unwanted adipose tissue and for tumor ablation [47,48]. The effects of HIFU application to tissue depend on the exposure time and the local temperature achieved in the beam-focused area [49]. Thermal ablation occurs at tissue temperatures of 60–85 °C; however, tissue temperatures of 40–45 °C can induce thermal stress, known as the hyperthermic effect [49,50], which is characterized by the induction of HSP expression [49]. Previously, our group reported that HIFU application to tissues increased HSP70 levels, resulting in decreased inflammatory cytokine expression [51]. Prior studies have further reported that hyperthermia alters Cav-1 levels [52].

Despite the link between HIFU and collagen synthesis and the potential for heat to modulate Cav-1 expression, whether HIFU modulates Cav-1 expression directly to promote increased collagen synthesis in the skin remains unknown.

We hypothesize that HIFU application decreases Cav-1 levels, leading to increased ERK1/2 phosphorylation and enhanced fibroblast proliferation in aging skin. Moreover, we predict that Cav-1 reduction decreases Cav-1 binding with p53 inhibitors MDM2 or Sirt1, releasing cells from cell cycle arrest, promoting fibroblast proliferation, and increasing collagen and elastin (ELN) fiber synthesis. In this study, we tested these hypotheses using an in vitro H_2_O_2_-induced cellular senescence model and an in vivo animal model of aging skin.

## 2. Materials and Methods

### 2.1. HIFU System

We used an HIFU system (LinearZ; Jeisys Medical Inc., Seoul, Republic of Korea) that produces thermal energy from the output of a focused ultrasound transducer with an operating frequency of 7 MHz. The system has two modes: LINEAR and DOT. The DOT mode generates focal energy points separated by defined distances, whereas the LINEAR mode generates linear energy without spaces between focal points. The maximum output energy of the system is 3.0 J. This study used the 7 MHz transducer in either the LINEAR or DOT mode at a 0.5 mm focal depth to deliver 0.3, 0.5, or 0.7 J of energy.

### 2.2. Temperature Measurements in the Dermal Layer after HIFU Treatment

Female porcine skin (6 months old) was used to measure dermal temperatures after HIFU treatment at 0.3, 0.5, or 0.7 J in the LINEAR or DOT mode. Skin samples at least 6 cm thick were prepared, and a thermometer (Fluoroptic^®^ Thermometer m3300 Biomedical Lab Kit; Luxtron Corp, Santa Clara, CA, USA) was inserted into the dermis at the transducer’s focal depth (0.5 mm). The test sample was immersed in a hot water tank until it reached human body temperature (35–37 °C). Ultrasound gel (Sanipia Inc., Gimje, Republic of Korea) was applied to the sample surface, which was then placed into contact with the HIFU cartridge. HIFU energy was delivered in a single application, and the peak temperature of the focused energy was measured.

### 2.3. Thermal Pattern Confirmation after HIFU Treatment

Female porcine skin was used to confirm the thermal pattern following HIFU treatment at 0.7 J, delivered at a consistent focal depth of 4.5 mm in the LINEAR or DOT mode. Porcine skin tissues at least 4.5 mm thick were pre-immersed in a water tank at a temperature of 35–37 °C, like human body temperature. Room-temperature ultrasound gel (Sanipia Inc.) was applied to the sample surface, which was then placed into contact with the HIFU cartridge. Thermal images were captured using a thermal imaging infrared camera (FLIR C3-X; FLIR, Wilsonville, OR, USA). Using the same energy and depth settings, the LINEAR mode yields a larger thermal area, whereas the DOT mode exhibits a more concentrated thermal pattern.

### 2.4. In Vitro Model for Testing the Effects of HIFU Treatment

Human fibroblasts (CCD-986Sk; American Type Culture Collection, Manassas, VA, USA) were cultured in growth medium (Iscove’s modified Dulbecco’s medium; Welgene, Gyeongsan, Republic of Korea) supplemented with 10% fetal bovine serum (Gibco^TM^, Thermo Fisher Scientific, Rockford, IL, USA) and 1% penicillin/streptomycin (Welgene) at 37 °C in an atmosphere containing 5% CO_2_. To induce fibroblast senescence, the cells were treated with 350 μM H_2_O_2_ (Sigma-Aldrich, St. Louis, MO, USA) for 1.5 h, washed with Dulbecco’s phosphate-buffered saline (Gibco^TM^, Thermo Fisher Scientific), and cultured in fresh growth medium for 72 h [53]. HIFU (LINEAR or DOT mode; 7 MHz frequency; 0.3, 0.5, or 0.7 J of energy) was applied, followed by incubation for an additional 48 h, after which cell lysates and supernatants were harvested for protein expression analyses.

### 2.5. Cav-1 Silencing in Senescent Fibroblasts

To investigate the role of Cav-1 in the effectiveness of HIFU treatment, Cav-1 gene expression was suppressed in senescent fibroblasts prepared as in Section 2.4. The senescent fibroblast at 70–80% confluence was transfected with Cav-1–targeting short interfering RNA (siRNA; Santacruz Biotechnology Technology, Dallas, TX, USA) using Lipofectamine 3000 reagent (Invitrogen, Waltham, MA, USA), according to the manufacturer’s protocol. Briefly, 1.5 μL Lipofectamine 3000 reagent, 500 ng Cav-1 shRNA, and 2 μL P3000 reagent were mixed in 100 μL of a serum-free medium to generate the DNA–lipid complex. The DNA–lipid complex was incubated at room temperature for 15 min and then diluted in the serum-free medium and cultured with fibroblasts (CCD986-Sk) at 37 °C, in an atmosphere containing 5% CO_2_ for 24 h. After 24 h, the cells were treated with HIFU, as described in Section 2.4.

### 2.6. Application of Hyperthermia to Senescent Fibroblasts

To confirm the correlation between HIFU treatment and thermal effects, fibroblast senescence was induced, as described in Section 2.4. Senescent fibroblasts were exposed to temperatures equivalent to those generated by the HIFU system for 1 min, followed by incubation for 48 h at 37 °C in an atmosphere containing 5% CO_2_. After 48 h, cell lysates were collected for RNA expression analyses.

### 2.7. Aging Mouse Model to Test the Effects of HIFU Treatment on Skin Rejuvenation

Female C57BL/6 mice (8 weeks old and 11 months, 3 weeks old) were purchased from Orient Bio (Seongnam, Republic of Korea) and allowed to acclimatize for 1 week. Mice were housed at a controlled temperature of 22 ± 5 °C and a relative humidity of 50 ± 10%, with a 12 h light/dark cycle. All mice had ad libitum access to a standard laboratory diet and water throughout the experiment. After a 1-week acclimation period, the 9-week-old mice were classified as the young group for the control, and the 12-month-old mice were classified as the aging group. Mice in the aging group were randomly divided into the following seven groups: aging control, aging/LINEAR 0.3 J, aging/LINEAR 0.5 J, aging/LINEAR 0.7 J, aging/DOT 0.3 J, aging/DOT 0.5 J, and aging/DOT 0.7 J.

HIFU was applied to aging mice in the experimental groups using the HIFU system to assess the effects on skin rejuvenation [51]. Mice were applied with ultrasound gel (Sanipia Inc.), and treated with HIFU in LINEAR or DOT mode, as indicated, with a 7 MHz frequency, 0.5 mm focal depth (in the dermis), and energy at 0.3, 0.5, or 0.7 J. HIFU was applied to the dorsal dermis of all mice in the aging/LINEAR 0.3 J, aging/LINEAR 0.5 J, aging/LINEAR 0.7 J, aging/DOT 0.3 J, aging/DOT 0.5 J, and aging/DOT 0.7 J groups. Mice in the young and aging control groups received no treatment other than the ultrasound gel. Four weeks following a one-time application of the HIFU, mice were anesthetized via inhalation of 3% isoflurane (HANA Pharm Co., Ltd., Seoul, Republic of Korea) and 1.5% O_2_, the HIFU treated areas were shaved, and skin tissues were collected for analysis. All animal welfare and experimental protocols in this study were approved by the Center of Animal Care and Use Animal Center Ethics Board and were executed humanely in accordance with the Institutional Animal Care and Use Committee (IACUC) of Gachon University and guidelines of the Association for Assessment and Accreditation of Laboratory Animal Care (IACUC approval number: LCDI-2022-0106). 

### 2.8. Protein Isolation

Cells were washed with phosphate-buffered saline (Sigma-Aldrich) and a scraper (SPL, Seoul, Republic of Korea) was used to scrape cells into a RIPA lysis buffer supplemented with protease and phosphatase inhibitors (EzRIPA buffer kit; ATTO Corporation, Tokyo, Japan). Skin tissues were homogenized in the RIPA lysis buffer supplemented with protease and phosphatase inhibitors using a bead homogenizer (Allsheng Instrument, Hangzhou, China) at 6.0 m/s for five cycles of 40 s with 45 s intervals. After homogenization, homogenates were incubated on ice for 10 min to facilitate cell lysis and protein solubilization. Following the initial lysis and homogenization step, cell and tissue samples were sonicated (high power, resting time 1 min, working time 10 s; CosmoBio Co., Ltd., Tokyo, Japan) and centrifuged at 14,000× *g* for 15 min at 4 °C to separate the soluble protein fraction from insoluble debris and organelles. The concentration of the soluble protein fraction was measured using a bicinchoninic acid assay kit (BCA kit; Thermo Fisher Scientific).

### 2.9. Western Blot

To prepare cell lysates and skin tissue samples, 30 µg of soluble protein was mixed with sample buffer (4× LDS buffer; Thermo Fisher Scientific) and a 10× reducing agent (Thermo Fisher Scientific). The protein samples were then heated at 70 °C for 10 min to denature proteins. To verify Cav-1 protein expression, denatured protein samples were electrophoresed at 200 V for 25 min through a 12% SDS-polyacrylamide gel in a MOPS buffer (Invitrogen, Waltham, MA, USA). To verify the expression of all other proteins, denatured protein samples were electrophoresed at 200 V for 25 min through 10% SDS-polyacrylamide gels in a MOPS buffer. Separated proteins were transferred to polyvinylidene fluoride (PVDF) membranes (Merck Millipore, Burlington, MA, USA) using a semi-dry transfer system (ATTO Corporation) at 1 A for 10 min. To prevent nonspecific binding, PVDF membranes were incubated with 5% skim milk (LPS solution, Daejeon, Republic of Korea) at room temperature for 1 h. After rinsing with Tris-buffered saline containing 0.1% Tween 20 (TTBS; LPS solution), PVDF membranes were incubated with appropriately diluted primary antibodies (Table S1) at 4 °C overnight. Following three washes with TTBS, membranes were incubated with horseradish peroxidase (HRP)-conjugated secondary antibodies (Vector Laboratories, Burlingame, CA, USA) at room temperature for 1 h. Protein bands were visualized using an enhanced chemiluminescence solution (CytivaTM, Marlborough, MA, USA) and imaged using a ChemiDoc Imaging System (Bio-Rad, Hercules, CA, USA). Protein bands were quantified using Image J 1.53 software from the National Institutes of Health (NIH, Bethesda, MD, USA) [54] and normalized to quantified beta-actin bands to control for differences in protein loading. Protein expression values in experimental samples derived from aging mice in a single blot are expressed relative to the mean expression value of the first bar.

### 2.10. Co-Immunoprecipitation (Co-IP)

Washed cells were scraped into prepared NP buffer (GeneDEPOT, Katy, TX, USA) containing protease and phosphatase inhibitors (ATTO Corporation). Skin tissues (100 mg) were homogenized in NP buffer containing protease and phosphatase inhibitors (ATTO Corporation) using a bead homogenizer (Allsheng Instrument) at 6.0 m/s for five cycles of 40 s with 45 s intervals. Homogenized samples were incubated on ice for 10 min, sonicated (high power, resting time 1 min, working time 10 s; CosmoBio Co., Ltd.), and centrifuged at 14,000× *g* for 15 min at 4 °C. Soluble protein concentrations were measured using a BCA kit (Thermo Fisher Scientific) following the manufacturer’s instructions. For pre-clearance, proteins were incubated with 10 μL A/G agarose beads (GeneDEPOT) for 1 h, at 4 °C with gentle rotation (KBT, Seongnam, Republic of Korea). Subsequently, samples were centrifuged at 2500 rpm for 15 min at 4 °C to remove beads and proteins with nonspecific binding. The precleared protein sample (1 mg) was incubated with 1 µg Cav-1 primary antibody (FineTest, Wuhan, China) overnight at 4 °C with gentle rotation. Protein A/G agarose beads were added to the mixture and incubated overnight at 4 °C with gentle rotation to capture the antibody–protein complex, followed by centrifugation at 2500 rpm for 15 min at 4 °C. The supernatant was discarded, and the pellet was washed in cold NP buffer to remove any nonspecifically bound proteins. Proteins were eluted through the addition of 4× LDS sample buffer (Thermo Fisher Scientific) and a 10× sample reducing agent (Thermo Fisher Scientific) to the beads, followed by incubation at 70 °C for 10 min. Samples were centrifugated at 2500 rpm for 15 min at 4 °C. Samples were evaluated by Western blot as described in Section 2.9.

### 2.11. Enzyme-Linked Immunosorbent Assay (ELISA)

The coating solution was prepared by dissolving 0.6% sodium bicarbonate and 0.3% sodium carbonate (Sigma-Aldrich) in distilled water. The prepared coating solution mixture was applied to 96-well plates and incubated overnight at 4 °C to ensure proper coating. The plates were then washed with phosphate-buffered saline containing 0.1% tween 20 (TPBS). The 96-well plates coated with the mixture were loaded with 5% skim milk. The plates were then incubated at room temperature for 6 h to block non-specific protein binding. Following the protein-blocking step, the plates were washed thoroughly with TPBS to remove unbound skim milk and reduce background noise. The human fibroblast supernatant (collected in Section 2.4 and Section 2.5) was added and incubated at 4 °C overnight to immobilize proteins onto the plate surface. After washing with TPBS to remove unbound proteins, primary antibodies (Table S1) were added to the wells and incubated at 4 °C overnight to allow for specific binding to the immobilized proteins. After rinsing with TPBS, HRP-conjugated antibodies (Vector Laboratories) were added to the wells and incubated at room temperature for 2 h to binding to the primary antibodies. Unbound HRP-conjugated antibodies were washed out with TPBS, and 3,3′,5,5′-tetramethylbenzidine (Sigma-Aldrich) was added to the wells. The plates were incubated for 10 min to develop the color signal. The color development reaction was stopped by adding 2 M H_2_SO_4_. The absorbance of each well was measured at 450 nm using an ELISA plate reader (Multiskan SkyHigh Photometer; Thermo Fisher Scientific).

### 2.12. Hydroxyproline Assay

Hydroxyproline levels in collected supernatants of fibroblasts (obtained as described in Section 2.4 and Section 2.5) and skin tissue samples (obtained as described in Section 2.6) were measured using a hydroxyproline ELISA kit (Aviva Systems Biology, San Diego, CA, USA). Briefly, samples and standards were loaded into each well of a microplate pre-coated with hydroxyproline, followed by the addition of a 1× hydroxyproline–biotin complex to each well and incubation for 1 h at 37 °C. After washing with the 1× wash buffer, 1× avidin–HRP conjugate was added to each well and incubation was conducted again for 45 min at 37 °C. After rinsing with a 1× wash buffer, a 3,3′,5,5′-tetramethylbenzidin substrate was added, followed by incubation for 15 min at 37 °C in the dark to allow for color development. After color developed, a stop solution was added, and optical density was measured at 450 nm using a microplate reader (Multiskan SkyHigh Photometer; Thermo Fisher Scientific).

### 2.13. Paraffin-Embedded Skin Tissue Block Preparation and Sectioning 

Skin tissues were fixed in cold 4% paraformaldehyde (Sigma-Aldrich) for 48 h. Following fixation, the tissues were dehydrated by immersion in increasing concentrations of ethanol. Subsequently, the tissues were cleared in xylene and embedded in paraffin using a tissue processor (Sakura Seiki Co., Ltd., Tokyo, Japan). The paraffin-embedded tissue blocks were sectioned into 7 µm thick slices using a microtome (Thermo Fisher Scientific). Obtained tissue sections were air-dried and placed in a 60 °C oven overnight to enhance tissue adhesion. Skin tissue sections were deparaffinized and rehydrated by sequential immersion in xylene (Duksan) and a gradient of 70–100% ethanol (Duksan).

### 2.14. 3,3′-Diaminobenzidine (DAB) Staining

The skin tissue samples, prepared according to the procedure outlined in Section 2.13, underwent deparaffinization and rehydration. This step involved a stepwise incubation process, starting with a series of xylene (Duksan) treatments followed by a gradient of alcohols (Duksan) ranging from 70–100%. To facilitate antigen retrieval, the skin tissue sections were subjected to a 20 s microwave boiling in a sodium citrate buffer (pH 6.0), then cooled in distilled water. After a PBS wash, the sections were treated with a 1% bovine serum solution for 10 min at room temperature to block nonspecific binding. Subsequently, the skin tissue sections were incubated overnight at 4 °C with anti-Collagen type I (1:50; Santa Cruz Biotechnology, Dallas, TX, USA) and an anti-Collagen type III antibody (1:100; Bioss, Woburn, MA, USA) (listed in Table S1). After another round of PBS washing, the slides were exposed to biotinylated secondary antibodies (1:200; Vector Laboratories) for 1 h at room temperature. After 30 min application of an ABC kit (Vector Laboratories), to achieve visual color development, the washed tissue sections were treated with a DAB solution (Sigma-Aldrich) for 15 min, resulting in a brown coloration. For counterstaining, the tissue sections were subjected to a 30 s incubation with hematoxylin (Korea Pathology Technical Center, Cheong Ju, Republic of Korea) and rinsing with distilled water. Dehydration was carried out using graded alcohols (70~100%) and xylene. The final step involved mounting the sections using a DPX mounting solution (Sigma-Aldrich). The stained tissues were imaged using a slide scanner (Motic Scan Infinity 100; Motic, Beijing, China). The intensity of Collagen types I and III was quantified using the ImageJ 1.53 software (NIH).

### 2.15. RNA Extraction and cDNA Synthesis

RNA was extracted from cell lysates (1 × 10^6^ cells per mL) using an RNAiso reagent (TAKARA, Tokyo, Japan), following the manufacturer’s instructions. In brief, lysed samples were combined with chloroform (Samchun, Seoul, Republic of Korea) and centrifugation was performed at 12,000× *g* for 15 min at 4 °C to separate the RNA-containing aqueous phase, which was carefully transferred to a new tube. RNA was precipitated by the addition of isopropanol (Duksan, Seoul, Republic of Korea), followed by incubation for 10 min at room temperature. Precipitated RNA was pelleted by centrifugation at 12,000× *g* for 10 min at 4 °C and washed with 75% cold ethanol (Sigma-Aldrich). After drying for 10 min at room temperature, the RNA pellet was dissolved in diethyl-pyrocarbonate-treated water (DEPC water; Biosesang, Seongnam, Republic of Korea). RNA concentration and purity were determined using a NanoDrop spectrophotometer (Thermo Fisher Scientific).

For cDNA synthesis, 1 µg of extracted RNA was mixed with Oligo DT primers (TAKARA) and dNTPs (TAKARA) in RNase-free distilled water (TAKARA) and incubated at 65 °C for 5 min. The mixture was then combined with reverse transcriptase (TAKARA) and RNase inhibitor (TAKARA), followed by incubation at 42 °C for 45 min and at 95 °C for 5 min, using a thermal cycler (Bio-Rad).

### 2.16. Quantitative Real-Time Polymerase Chain Reaction (qRT-PCR) 

For qRT-PCR, a reaction mixture containing 5 µL ROX plus SYBR green premix (TAKARA), 0.8 µL each of reverse and forward primer (Table S2), 1.7 µL distilled water, and 2.5 µL cDNA template was prepared, resulting in a total volume of 10 µL. Amplification and melting curve analyses were conducted using a real-time PCR instrument (Thermo Fisher Scientific). The qRT-PCR amplification protocol consisted of an initial denaturation step at 95 °C for 10 min, followed by 40 cycles of denaturation at 95 °C for 15 s, annealing and extension at 60 °C for 1 min, and a final denaturation step at 95 °C for 15 s. Following amplification, a melting analysis was performed by gradually increasing the temperature from 60 °C to 95 °C at a rate of 0.075 °C/s. To determine gene expression levels, the comparative CT method (ΔΔCT) was employed. All mRNA levels were normalized to the expression level of actin beta (ACTB) and are expressed relative to the level for the first bar of each graph.

### 2.17. Masson’s Trichrome Staining

To stain collagen fibers, skin tissues were incubated in Bouin solution (Scytek Laboratories, West Logan, UT, USA) at 60 °C for 1 h, followed by washing with distilled water. Sections were then sequentially placed in iron hematoxylin (Scytek Laboratories) for 10 min, Biebrich scarlet acid fuchsin solution (Scytek Laboratories) for 1 min, phosphomolybdic-phosphotungstic acid solution (Scytek Laboratories) for 15 min, and aniline blue solution (Scytek Laboratories) for 3 min at room temperature. Following staining, slides were dehydrated using graded alcohols (70–100%), cleared in xylene, mounted with DPX mount solution (Sigma-Aldrich), and examined under an optical microscope (Olympus, Tokyo, Japan) equipped with a slide scanner (Motic, Vancouver, BC, Canada). Collagen fiber density analysis was performed on all images using ImageJ software (NIH) [55].

### 2.18. Herovici’s Collagen Staining

Mature collagen fibers and newly synthesized collagen fibers were identified in skin tissues using a Herovici Collagen Stain Kit (Scytek Laboratories) [56,57]. Deparaffinized slides were incubated in Weigert’s iron hematoxylin for 8 min to stain nuclei, followed by rinsing with tap water and distilled water. After rinsing, slides were treated with Herovici solution for 2 min, washed, dehydrated using graded alcohols (70–100%), cleared in xylene, and mounted with DPX mounting solution (Sigma-Aldrich). Microscopic observation and imaging were conducted using a slide scanner (Motic). Newly synthesized collagen fibers were stained blue, and mature collagen fibers were stained red. The staining densities were quantitatively analyzed using ImageJ software (NIH) [58].

### 2.19. Verhoeff’s Elastic Staining

To stain elastic fibers, deparaffinized skin tissues were exposed to an elastic staining solution (Scytek Laboratories) at room temperature for 15 min, followed by rinsing with tap water. After rinsing, slides were incubated with 20 drops of 2% ferric chloride differentiation solution (Scytek Laboratories), washed with distilled water, dehydrated using graded alcohols (70–100%), cleared in xylene, and mounted using DPX mount solution (Sigma-Aldrich). Mounted slides were examined under an optical microscope equipped with a slide scanner (Motic) to confirm staining. Captured images were analyzed for elastic fiber densities using ImageJ software (NIH) [59].

### 2.20. Measurement of Epidermal Thickness

To measure epidermal thickness, the deparaffinized skin tissues were stained with hematoxylin solution (Korea Pathology Technical Center, Cheongju, Republic of Korea) for 1 min and rinsed in running tap water for 3 min. Afterward, the sections were briefly exposed to ammonia water (Samchun) for 10 s, immersed in eosin solution (Korea Pathology Technical Center) for 1 min, and washed with running water. Stained sections were then dehydrated using graded alcohols (70–100%), cleared in xylene, and mounted using DPX mount solution (Sigma-Aldrich). Stained slide images were captured using a slide scanner (Motic). To determine epidermal thickness, ImageJ software (NIH) was used to measure epidermal thickness in five sections per sample. Data are expressed as the average epidermal thickness per sample.

### 2.21. Statistical Analysis

Data were validated by performing at least three replicates for each experiment (*n* = 3), and the results are presented as the mean ± standard deviation. The Kruskal–Wallis test was used to compare between groups, and the Mann–Whitney U test was used for post hoc analysis, with *p* < 0.05 considered significant. All statistical analyses were performed in SPSS v.22 (IBM Corporation; Armonk, NY, USA).

## 3. Results

### 3.1. HIFU Increases Local Skin Temperatures

To precisely evaluate changes in skin temperature in response to HIFU, we applied HIFU in the LINEAR and DOT modes to ex vivo pig skin and measured dermal temperatures. We found that when HIFU was applied in LINEAR mode at 0.3, 0.5, and 0.7 J, dermal temperatures increased to 42.48 ± 0.19 °C, 48.32 ± 0.23 °C, and 52.30 ± 0.37 °C, respectively (Table S3 and Figure S1). When HIFU was applied in DOT mode, dermal temperatures increased to 42.64 ± 0.34 °C, 49.94 ± 0.47 °C, and 54.52 ± 0.36 °C at 0.3, 0.5, and 0.7 J, respectively. No significant difference in dermal temperatures was observed between both LINEAR and DOT modes at 0.3 J. However, at 0.5 and 0.7 J, dermal temperatures in response to the LINEAR mode were significantly lower than those in response to the DOT mode (Table S3 and Figure S1).

**Figure 1:**
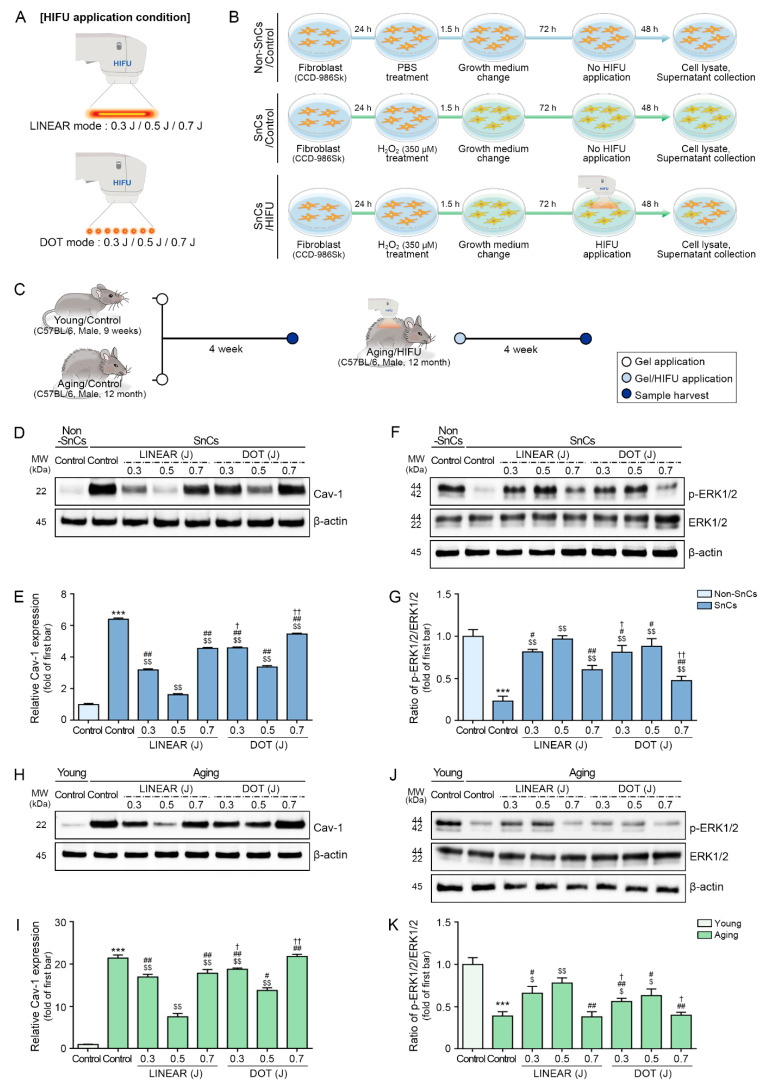
Effect of HIFU application on Cav-1 expression and of the ratio of p-ERK1/2 to total ERK1/2 in senescent fibroblasts and the skin of aging mice. (**A**) This diagram shows the conditions of the HIFU application used in this study. (**B**) Schematic diagram demonstrating how HIFU efficacy was evaluated in vitro in human fibroblasts. Fibroblasts (CCD-986Sk) were treated with H_2_O_2_ (350 μM) for 1.5 h to induce senescence and cultured in growth medium for 72 h, followed by HIFU application (LINEAR or DOT modes at 0.3, 0.5, or 0.7 J). At 48 h after HIFU application, cell lysates were collected. Non-SnCs and control SnCs were collected without HIFU application. (**C**) Schematic diagram depicting the animal experiments performed in this study. HIFU was applied in LINEAR or DOT modes at 0.3, 0.5, or 0.7 J to the back skin of aging animals, and skin samples were obtained 4 weeks after HIFU application. For young and aging control mice, skin was obtained 4 weeks after the start of the experiment with only gel applied and no HIFU application. (**D**) Cav-1 protein expression in fibroblasts was evaluated by Western blot. (**E**) Quantification of Cav-1 expression in fibroblasts, as evaluated by Western blot. (**F**) Total ERK1/2 and p-ERK1/2 protein expression in fibroblasts was evaluated by Western blot. (**G**) Quantification of the p-ERK1/2 to total ERK1/2 ratio in fibroblasts, as evaluated by Western blot. (**H**) Cav-1 protein expression in skin tissues were evaluated by Western blot. (**I**) Quantification of Cav-1 expression in skin tissues, as evaluated by Western blot. (**J**) Total ERK1/2 and p-ERK1/2 protein expression in skin tissues was evaluated by Western blot. (**K**) Quantification of the p-ERK1/2 to total ERK1/2 ratio in skin tissues, as evaluated by Western blot. The band intensities of single blots were quantified, and expression levels are reported relative to those indicated by the first bar of the graph after normalization to β-actin levels (loading control). Data were validated with at least three replicates for each experiment (*n* = 3) and are presented as the mean ± standard deviation. ***, *p* < 0.001 for first bar vs. second bar; $, *p* < 0.05, $$, *p* < 0.01 vs. second bar; #, *p* < 0.05, ##, *p* < 0.01 vs. fourth bar; †, *p* < 0.05, ††, *p* < 0.01 vs. seventh bar. Cav-1, caveolin-1; HIFU, high-intensity focused ultrasound; ERK1/2, extracellular signal-regulated kinase1/2; MW, molecular weight; Non-SnCs, non-senescent cells; p-ERK1/2, phosphorylated-ERK1/2; SnCs, senescent cells.

Although the DOT mode generates focal points separated by some distance, the LINEAR mode generates uninterrupted linear energy [30]. Despite both modes having the same energy output of 0.7 J, the LINEAR mode results in a larger thermal area, whereas the DOT mode exhibits a more concentrated thermal pattern with a higher peak temperature than the LINEAR mode (Figure 1A and Figure S2).

### 3.2. HIFU Decreases p16 and Increases HSP70 Expression

First, we evaluated whether HIFU application (LINEAR mode, 0.3 J, 0.5 J, or 0.7 J; DOT mode, 0.3 J, 0.5 J, or 0.7 J) alters Cav-1 expression levels in an in vitro model of fibroblast senescence in which human fibroblast (CCD-986Sk) cells were treated with H_2_O_2_ to induce cellular senescence [53] (Figure 1A,B). To confirm the induction of cellular senescence, we evaluated p16 expression. H_2_O_2_-induced senescent fibroblasts had significantly higher levels of p16 expression than normal control fibroblasts (non-senescent fibroblasts). We further evaluated whether HIFU affects p16 expression in H_2_O_2_-induced senescent fibroblasts. HIFU treatment of H_2_O_2_-induced senescent fibroblasts resulted in decreased p16 expression compared with untreated senescent fibroblasts. For both LINEAR and DOT modes, p16 expression was lowest when HIFU was applied at the 0.5 J level, and at 0.5 J, p16 expression was lower in response to the LINEAR mode than in response to the DOT mode (Figure S3A,B).

HSP70 expression levels in H_2_O_2_-induced senescent fibroblasts were lower than the levels in normal control fibroblasts. In senescent fibroblasts, HSP70 expression was higher following HIFU treatment than in untreated cells. In both LINEAR and DOT modes, HSP70 expression levels were highest in response to HIFU delivered at the 0.7 J level. HSP70 expression in response to the LINEAR mode was lower than that in response to DOT mode at 0.7 J (Figure S3C,D). We then applied HIFU to the dorsal skin of 9-week-old (young) and 12-month-old (aging) mice in either LINEAR or DOT mode at the 0.3, 0.5, or 0.7 J energy levels (Figure 1A,C). Skin was harvested 4 weeks after HIFU application, and p16 and HSP70 expression levels were evaluated. Expression of p16 was higher in skin from aging mice than in skin from young mice, and HIFU administration decreased p16 expression. In both LINEAR and DOT modes, p16 expression levels were the lowest in response to HIFU delivered at the 0.5 J level, and p16 expression in response to the LINEAR mode was lower than that in response to DOT mode at 0.5 J (Figure S3E,F).

HSP70 expression levels were lower in skin from aging mice than in skin from young mice, and expression increased in response to HIFU. In both LINEAR and DOT modes, HSP70 expression increased in response to HIFU application, and HSP70 expression in response to LINEAR mode was lower than that in response to the DOT mode at 0.7 J (Figure S3G,H).

### 3.3. HIFU Decreases Cav-1 and Increases ERK1/2 Expression

Cav-1 expression was higher in H_2_O_2_-induced senescent fibroblasts than in control fibroblasts. Application of HIFU to senescent fibroblasts decreased Cav-1 expression compared with untreated senescent fibroblasts. In both LINEAR and DOT modes, Cav-1 expression levels were lowest in response to HIFU delivered at the 0.5 J level. Cav-1 expression in response to the LINEAR mode was lower than that in response to the DOT mode at 0.5 J (Figure 1D,E).

The ratio of phosphorylated (p)-ERK1/2 to total ERK1/2 was lower in H_2_O_2_-induced senescent fibroblasts than in control fibroblasts. HIFU application to senescent fibroblasts increased the p-ERK1/2 to total ERK1/2 ratio compared with that in untreated senescent fibroblasts. In both LINEAR and DOT modes, the p-ERK1/2 to total ERK1/2 ratios were the highest in response to HIFU delivered at the 0.5 J level. The p-ERK1/2 to total ERK1/2 ratio in response to the LINEAR mode was higher than that in response to the DOT mode at 0.5 J (Figure 1F,G).

Cav-1 expression was higher in the skin of aging mice than in the skin of young mice. However, HIFU application to the skin of aging mice decreased Cav-1 levels, with the most prominent effect observed for mice treated with HIFU delivered at 0.5 J in LINEAR mode (Figure 1H,I). Western blot analysis revealed a lower p-ERK1/2 to total ERK1/2 ratio in the skin of aging mice than in the skin of young mice, but the application of HIFU to the skin of aging mice increased this ratio, with the most prominent increase observed for mice treated with HIFU delivered at 0.5 J in LINEAR mode (Figure 1J,K).

### 3.4. HIFU Decreases Binding of Cav-1 with MDM2 and Sirt1 and Decreases p53 and p21 Levels

**Figure 2:**
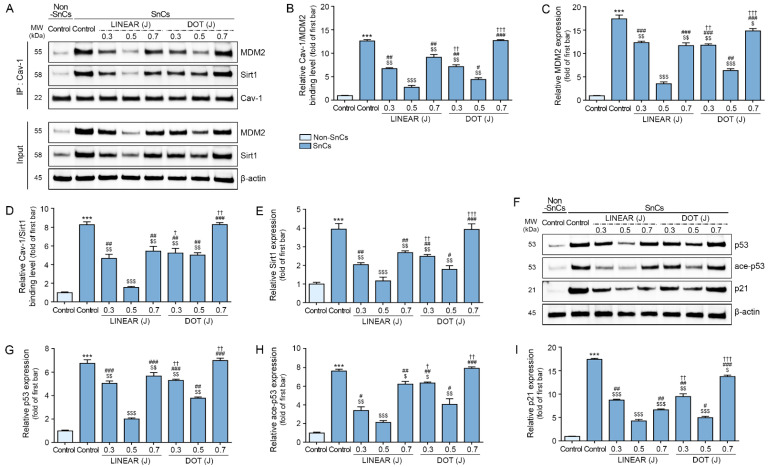
Reduced binding of Cav-1 with both MDM2 and Sirt1 and reduced levels of p53, p21, and ace-p53 in response to HIFU application to senescent fibroblasts. (**A**) Binding of Cav-1 with MDM2 and Sirt1 in fibroblasts, as measured by Co-IP. Total protein extracts of cells (Input) were immunoprecipitated with Cav-1. Protein–protein bindings were detected using MDM2 and Sirt1 by Western blot. (**B**–**E**) Quantification of Cav-1 binding with MDM2 in fibroblasts, as evaluated by Co-IP (**B**). Quantification of MDM2 in total protein extracts of fibroblasts (Input) was evaluated by Western blot (**C**). Quantification of Cav-1 binding with Sirt1 in fibroblasts, as evaluated by Co-IP (**D**). Quantification of Sirt1 in total protein extracts of fibroblasts (Input) was evaluated by Western blot (**E**). (**F**) The p53, ace-p53, and p21 protein expression in fibroblasts, as evaluated by Western blot. (**G**–**I**) Quantification of p53 (**G**), ace-p53 (**H**), and p21 (**I**) levels in fibroblasts, as evaluated by Western blot. The band intensities of single blots were quantified, and expression levels are presented relative to those indicated by the first bar of the graph after normalization to β-actin or Cav-1 levels (loading controls). Data were validated with at least three replicates for each experiment (*n* = 3) and are presented as the mean ± standard deviation. ***, *p* < 0.001 for first bar vs. second bar; $, *p* < 0.05, $$, *p* < 0.01 $$$, *p* < 0.001 vs. second bar; #, *p* < 0.05, ##, *p* < 0.01, ###, *p* < 0.001 vs. fourth bar; †, *p* < 0.05, ††, *p* < 0.01, †††, *p* < 0.01 vs. seventh bar. ace-p53, acetylated-p53; Cav-1, caveolin-1; Co-IP, co-immunoprecipitation; HIFU, high-intensity focused ultrasound; MDM2, mouse double minute 2 homolog; MW, molecular weight; Non-SnCs, non-senescent cells; Sirt1, sirtuin 1; SnCs, senescent cells.

We measured binding between Cav-1 and MDM2 in control and senescent fibroblasts by Co-IP. Binding between Cav-1 and MDM2 was detected at higher levels in senescent fibroblasts than in control fibroblasts, and HIFU application decreased binding levels. In both LINEAR and DOT modes, binding between Cav-1 and MDM2 was detected at the lowest levels in response to HIFU applied at the 0.5 J level. Binding between Cav-1 and MDM2 was detected at lower levels in response to the LINEAR mode than in response to the DOT mode at 0.5 J (Figure 2A–C).

Binding between Cav-1 and Sirt1 was higher in senescent fibroblasts than in control fibroblasts, and HIFU application decreased binding. In both LINEAR and DOT modes, binding between Cav-1 and Sirt1 was detected at the lowest levels in response to HIFU applied at the 0.5 J level. Binding between Cav-1 and Sirt1 was lower in response to the LINEAR mode than in response to the DOT mode at 0.5 J (Figure 2A,D,E).

Expression of p53, acetylated (ace)-p53, and p21 was measured in control and senescent fibroblasts. The p53 and ace-p53 expression levels were higher in senescent fibroblasts than in control fibroblasts, and HIFU application decreased p53 and ace-p53 expression levels. In both LINEAR and DOT modes, the lowest p53 and ace-p53 levels were observed in response to HIFU applied at the 0.5 J level. The p53 and ace-p53 expression levels were lower in response to the LINEAR mode than in response to the DOT mode at 0.5 J (Figure 2F–H).

Expression of p21 was higher in senescent fibroblasts than in control fibroblasts, and HIFU application decreased p21 expression. In both LINEAR and DOT modes, the lowest p21 expression levels were observed in response to HIFU delivered at the 0.5 J level. The p21 expression in response to the LINEAR mode was lower than in response to the DOT mode at 0.5 J (Figure 2F,I).

**Figure 3:**
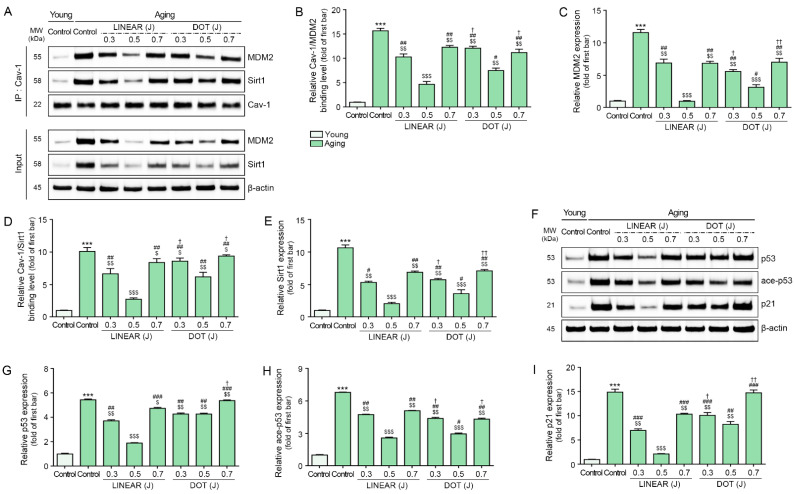
Reduced binding of Cav-1 with both MDM2 and Sirt1 and reduced levels of p53, p21, and ace-p53 in response to HIFU application to the skin of aging mice. (**A**) Binding of Cav-1 with MDM2 and Sirt1 in skin tissues, as measured by Co-IP. Total protein extracts of cells (Input) were immunoprecipitated with Cav-1. Protein–protein bindings were detected using MDM2 and Sirt1 by Western blot. (**B**–**E**) Quantification of Cav-1 binding with MDM2 in skin tissues, as evaluated by Co-IP (**B**). Quantification of MDM2 in total protein extracts of skin tissues (Input) was evaluated by Western blot (**C**). Quantification of Cav-1 binding with Sirt1 in skin tissues, as evaluated by Co-IP (**D**). Quantification of Sirt1 in total protein extracts of skin tissues (Input) was evaluated by Western blot (**E**). (**F**) The p53, ace-p53, and p21 protein expression in skin tissues, as evaluated by Western blot. (**G**–**I**) Quantification of p53 (**G**), ace-p53 (**H**), and p21 (**I**) levels in skin tissues, as evaluated by Western blot. The band intensities of single blots were quantified, and expression levels are presented relative to those indicated by the first bar of the graph after normalization to β-actin or Cav-1 levels (loading controls). Data were validated with at least three replicates for each experiment (*n* = 3) and are presented as the mean ± standard deviation. ***, *p* < 0.001 for first bar vs. second bar; $, *p* < 0.05, $$, *p* < 0.01 $$$, *p* < 0.001 vs. second bar; #, *p* < 0.05, ##, *p* < 0.01, ###, *p* < 0.001 vs. fourth bar; †, *p* < 0.05, ††, *p* < 0.01 vs. seventh bar. ace-p53, acetylated-p53; Cav-1, caveolin-1; Co-IP, co-immunoprecipitation; HIFU, high-intensity focused ultrasound; MDM2, mouse double minute 2 homolog; MW, molecular weight; Non-SnCs, non-senescent cells; Sirt1, sirtuin 1.

We next measured binding between MDM2 and Cav-1 in skin from young and aging mice. The results show binding was detected at higher levels in skin from aging mice than in skin from young mice. However, binding decreased in animals treated with HIFU, with the most prominent effect observed in mice receiving HIFU delivered at 0.5 J in LINEAR mode (Figure 3A–C). Similarly, we detected higher levels of binding between Sirt1 and Cav-1 in skin from aging mice than in skin from young mice, with decreased binding in response to HIFU treatment. The most prominent effect was detected in mice that received HIFU at 0.5 J in LINEAR mode (Figure 3A,D,E).

We then evaluated the expression of p53, ace-p53, and p21 in skin from young and aging mice by Western blot and found higher levels of all three in the skin of aging mice than in the skin of young mice. Consistent with our above findings, HIFU application decreased p53, ace-p53, and p21 levels, with the most prominent effect observed in response to HIFU delivered at 0.5 J in LINEAR mode (Figure 3F–I).

### 3.5. HIFU Application Decreases Cell Cycle Arrest and Increases Cell Proliferation

**Figure 4:**
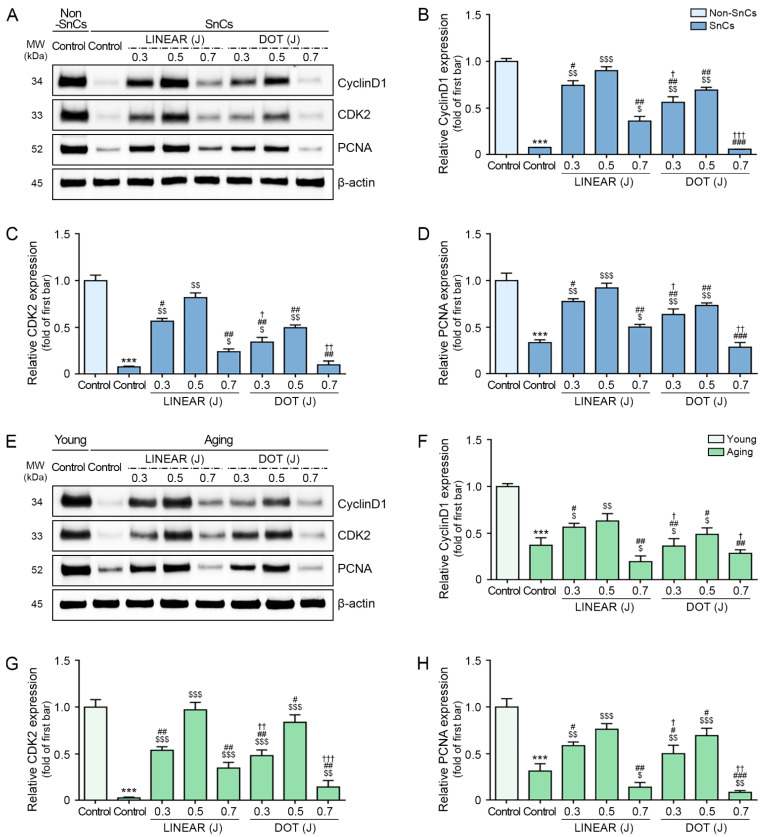
Activation of the cell cycle by HIFU application. (**A**) Cyclin D1, CDK2, and PCNA protein levels were assessed in fibroblasts by Western blot. (**B**–**D**) Quantification of data in panel A. (**E**) Cyclin D1, CDK2, and PCNA protein levels in skin tissues, as evaluated by Western blot. (**F**–**H**) Quantitative graphs of the data in panel (**E**). The band intensities of single blots were quantified, and expression levels are presented relative to those indicated by the first bar of the graph after normalization to β-actin levels (loading control). Data were validated with at least three replicates for each experiment (*n* = 3) and are presented as the mean ± standard deviation. ***, *p* < 0.001 for first bar vs. second bar; $, *p* < 0.05, $$, *p* < 0.01, $$$, *p* < 0.001 vs. second bar; #, *p* < 0.05, ##, *p* < 0.01, ###, *p* < 0.001 vs. fourth bar; †, *p* < 0.05, ††, *p* < 0.01, †††, *p* < 0.001 vs. seventh bar. CDK2, cyclin-dependent kinase 2; HIFU, high-intensity focused ultrasound; MW, molecular weight; Non-SnCs, non-senescent cells; PCNA, proliferating cell nuclear antigen; SnCs, senescent cells.

Cyclin D1 and CDK2 expression levels were lower in senescent fibroblasts than in control fibroblasts, and the expression of both proteins increased in response to HIFU application. In both LINEAR and DOT modes, the highest cyclin D1 and CDK2 expression levels were detected in response to HIFU delivered at the 0.5 J level. Cyclin D1 and CDK2 expression levels were higher in response to the LINEAR mode than in response to the DOT mode at 0.5 J (Figure 4A–C).

Based on these observations, we evaluated cell proliferation in normal and senescent fibroblasts by measuring proliferating cell nuclear antigen (PCNA) expression, which was lower in senescent fibroblasts than in control fibroblasts and increased in response to HIFU application. In both LINEAR and DOT modes, the highest PCNA expression levels were detected in response to HIFU delivered at the 0.5 J level. PCNA expression was higher in response to the LINEAR mode than in response to the DOT mode at 0.5 J (Figure 4A,D).

Cyclin D1 and CDK2 expression levels were lower in skin from aging mice than in skin from young mice. However, these levels increased in skin from aging mice treated with HIFU compared with untreated skin, with the most prominent increases observed in response to HIFU applied at 0.5 J in LINEAR mode (Figure 4E–G).

Consistent with our above findings, we found lower PCNA expression in the skin of aging mice compared with the skin of young mice, and PCNA expression increased in the skin of aging mice upon HIFU treatment compared with untreated skin. The most prominent increase in PCNA expression was observed in response to HIFU application at 0.5 J in LINEAR mode (Figure 4E,H).

### 3.6. HIFU Increases TIMP1 Expression and Decreases MMP1 Expression

We evaluated tissue inhibitor of metalloprotease 1 (TIMP1) and MMP1 expression in the supernatant of senescent fibroblast in vitro model via ELISA. MMP1 is the principal collagenase secreted from fibroblasts which can destroy fibrillar collagen types I, II, III, and IV [60]. 

**Figure 5:**
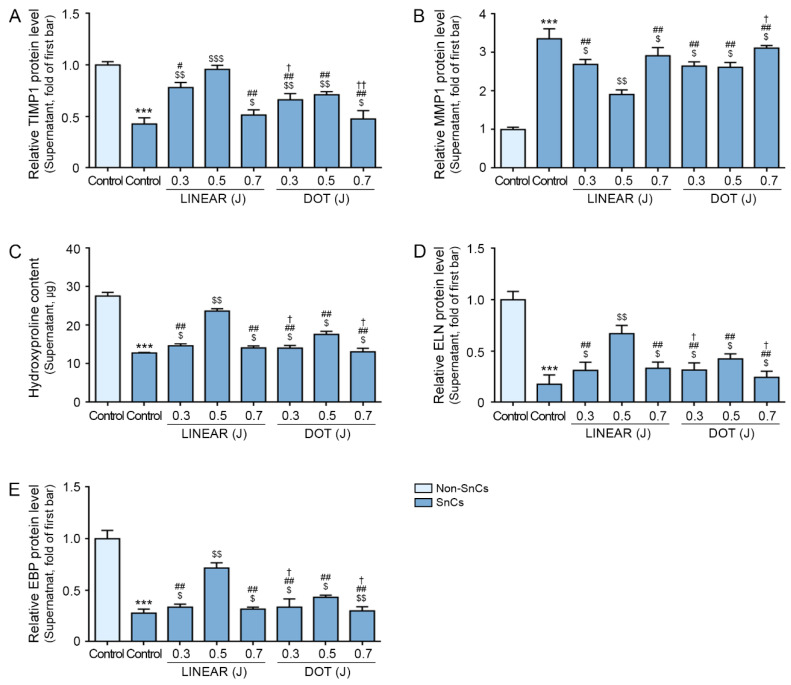
Effects of HIFU application on TIMP1, MMP1, hydroxyproline, ELN, and EBP levels in the supernatant of the senescent fibroblasts. (**A**,**B**) TIMP1 (**A**) and MMP1 (**B**) protein levels in the supernatant of SnCs were evaluated via ELISA. (**C**) Hydroxyproline contents in the supernatant of the SnCs were measured via hydroxyproline assay. (**D**,**E**) ELN (**D**) and EBP (**E**) protein levels in the supernatant of SnCs were evaluated via ELISA. The protein expression levels are presented relative to those indicated by the first bar in the graph. Data were validated with at least three replicates for each experiment (*n* = 3) and present the mean ± standard deviation. ***, *p* < 0.001 for first bar vs. second bar; $, *p* < 0.05, $$, *p* < 0.01, $$$, *p* < 0.001 vs. second bar; #, *p* < 0.05, ##, *p* < 0.01 vs. fourth bar; †, *p* < 0.05, ††, *p* < 0.01 vs. seventh bar. EBP, elastin-binding protein; ELISA, enzyme-linked immunosorbent assay; ELN, elastin; HIFU, high-intensity focused ultrasound; MMP1, matrix metalloproteinase 1; Non-SnCs, non-senescent cells; SnCs, senescent cells; TIMP 1, tissue inhibitor of metalloprotease 1.

TIMP1 expression levels were lower in senescent fibroblasts than in control fibroblasts, and the expression of TIMP1 increased in response to HIFU application. In both LINEAR and DOT modes, the highest TIMP1 expression levels were detected in response to HIFU delivered at the 0.5 J level. TIMP1 expression levels were higher in response to the LINEAR mode than the DOT mode at 0.5 J (Figure 5A). MMP1 expression levels were higher in senescent fibroblasts than in control fibroblasts, and the expression of MMP1 decreased in response to HIFU application. In both LINEAR and DOT modes, the lowest MMP1 expression levels were detected in response to HIFU delivered at the 0.5 J level. MMP1 expression levels were lower in response to the LINEAR mode than the DOT mode at 0.5 J (Figure 5B).

We measured the expression levels of hydroxyproline in the supernatant of senescent fibroblasts. Expression levels of hydroxyproline were lower in senescent fibroblasts than in control fibroblasts, and the expression of those proteins increased in response to HIFU application. In both LINEAR and DOT modes, the highest expression levels were detected in response to HIFU delivered at the 0.5 J level. Expression levels of hydroxyproline were higher in response to the LINEAR mode than in response to the DOT mode at 0.5 J (Figure 5C).

To further evaluate elastic fiber synthesis, we measured ELN and ELN-binding protein (EBP) levels in the supernatant of senescent fibroblasts. ELN and EBP expression levels were lower in senescent fibroblasts than in control fibroblasts, and the expression of those proteins increased in response to HIFU application. In both LINEAR and DOT modes, the highest expression levels were detected in response to HIFU delivered at the 0.5 J level. Expression levels of ELN and EBP were higher in response to the LINEAR mode than in response to the DOT mode at 0.5 J (Figure 5D,E).

**Figure 6:**
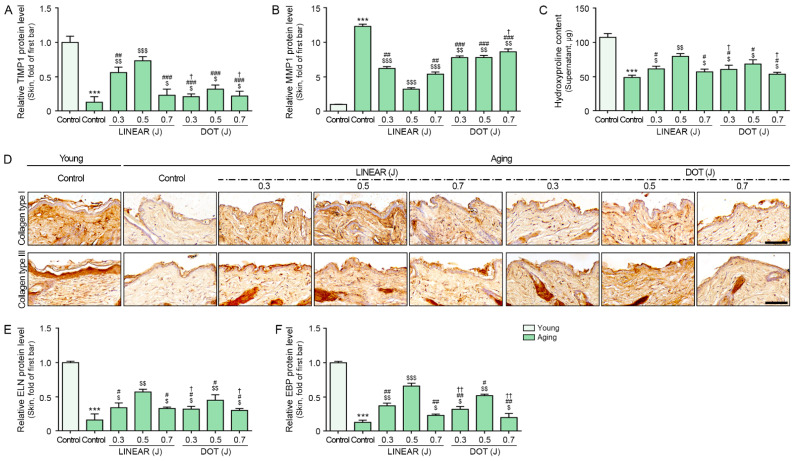
Effects of HIFU application on TIMP1, MMP1, collagen, hydroxyproline, and ELN levels in the skin of aging mice. (**A**,**B**) TIMP1 (**A**) and MMP1 (**B**) protein levels in skin tissues were evaluated via ELISA. (**C**) Hydroxyproline contents in skin tissues were measured via hydroxyproline assay. (**D**) Collagen types I and III were validated in skin tissues by DAB staining. Quantitative graphs can be found in Figure S4. (**E**,**F**) ELN (**E**) and EBP (**F**) protein levels in skin tissues were evaluated via ELISA. The protein expression levels are presented relative to those indicated by the first bar in the graph. Data were validated with at least three replicates for each experiment (*n* = 3) and present the mean ± standard deviation. ***, *p* < 0.001 for first bar vs. second bar; $, *p* < 0.05, $$, *p* < 0.01, $$$, *p* < 0.001 vs. second bar; #, *p* < 0.05, ##, *p* < 0.01, ###, *p* < 0.001 vs. fourth bar; †, *p* < 0.05, ††, *p* < 0.01 vs. seventh bar. EBP, elastin-binding protein; ELN, elastin; HIFU, high-intensity focused ultrasound; MMP1, matrix metalloproteinase1; TIMP 1, tissue inhibitor of metalloprotease 1.

TIMP1 expression in the aged skin was lower than that in the young skin. It was increased by HIFU and the most prominent response was shown when HIFU was delivered at 0.5 J in LINEAR mode. We detected elevated expression levels of MMP1 in the skin of aging mice compared with the skin of young mice. MMP1 expression levels decreased in HIFU-treated mice and most prominently in response to HIFU delivered at 0.5 J in LINEAR mode (Figure 6A,B).

We measured the expression levels of hydroxyproline and collagen type I and collagen type III for evaluating collagen synthesis. HIFU treatment increased the expression levels of hydroxyproline in the skin of aging mice compared with untreated skin, with the most prominent effects observed in response to HIFU delivered at 0.5 J in LINEAR mode (Figure 6C). HIFU treatment increased the expression levels of collagen type I and III in the skin of aging mice compared with untreated skin, with the most prominent effects observed in response to HIFU delivered at 0.5 J in LINEAR mode (Figure 6D and Figure S4).

To further evaluate elastic fiber synthesis, we measured ELN and ELN-binding protein (EBP) levels. Our results show that the levels of both were lower in skin from aging mice than in skin from young mice. Moreover, and consistent with our above observations, both ELN and EBP expression levels were higher in mice treated with HIFU than in untreated mice, with the most prominent effects observed in response to HIFU delivered at 0.5 J in LINEAR mode (Figure 6E,F).

### 3.7. HIFU Application Induces Decreased MMP Expression and Increased Collagen Synthesis via Cav-1

**Figure 7:**
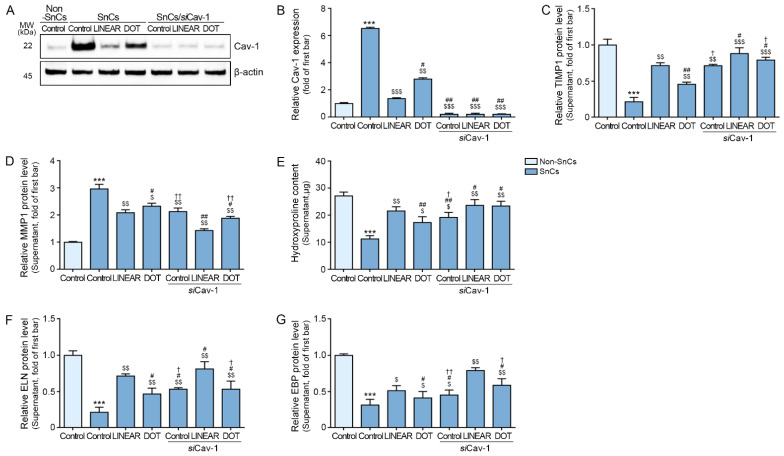
Association of Cav-1 expression with changes in TIMP1, MMP1 expression, collagen, and ELN synthesis in senescent fibroblasts treated with HIFU. (**A**) Cav-1 protein expression was evaluated in Cav-1-silenced fibroblasts by Western blot. (**B**) Quantification of panel 7A. (**C**,**D**) The TIMP1 (**C**) and MMP1 (**D**) protein levels in the supernatant of Cav-1-silenced fibroblasts were evaluated via ELISA. (**E**) Hydroxyproline contents in Cav-1-silenced fibroblasts were measured via hydroxyproline assay. (**F**,**G**) The ELN (**F**) and EBP (**G**) protein levels in the supernatant of Cav-1-silenced fibroblasts were evaluated via ELISA. The protein expression levels are presented relative to those indicated by the first bar in the graph. Data were validated with at least three replicates for each experiment (*n* = 3) and are presented as the mean ± standard deviation. Cav-1, caveolin 1; EBP, elastin-binding protein; ELISA, Enzyme-linked immunosorbent assay; ELN, elastin; HIFU, high-intensity focused ultrasound; MMP, matrix metalloproteinase; Non-SnCs, non-senescent cells; *si* Cav-1, silenced Cav-1; SnCs, senescent cells; TIMP 1, tissue inhibitor of metalloprotease 1. ***, *p* < 0.001 for first bar vs. second bar; $, *p* < 0.05, $$, *p* < 0.01, $$$, *p* < 0.001 vs. second bar; #, *p* < 0.05, ##, *p* < 0.01 vs. fourth bar; †, *p* < 0.05, ††, *p* < 0.01 vs. seventh bar.

To evaluate whether HIFU-induced increased TIMP1 and decreases in MMP expression are mediated by effects on Cav-1, we inhibited Cav-1 using an siRNA targeting Cav-1 (siCav-1) (Figure 7A,B).

TIMP1 expression was lower in senescent fibroblasts than in control fibroblasts, and expression levels increased following HIFU treatment. In Cav-1-silenced senescent fibroblasts, TIMP1 expression levels were higher than in senescent fibroblasts with normal Cav-1 expression. In Cav-1-silenced senescent fibroblasts, HIFU treatment increased TIMP1 expression by a smaller degree than HIFU treatment of senescent fibroblasts with normal Cav-1 expression (Figure 7C).

MMP1 expression was higher in senescent fibroblasts than in control fibroblasts, and expression levels decreased following HIFU treatment. In Cav-1-silenced senescent fibroblasts, MMP1 expression levels were lower than in senescent fibroblasts with normal Cav-1 expression. In Cav-1-silenced senescent fibroblasts, HIFU treatment decreased MMP1 expression by a more minor degree than HIFU treatment of senescent fibroblasts with normal Cav-1 expression (Figure 7D).

Hydroxyproline expression levels were lower in senescent fibroblasts than in control fibroblasts, and HIFU application increased the expression levels of these proteins. Cav-1 silencing also increased the expression levels of these proteins in senescent fibroblasts, with further increases observed upon HIFU application (Figure 7E).

ELN and EBP expression levels were lower in senescent fibroblasts than in control fibroblasts, and the expression of these proteins was increased as a result of HIFU treatment. Cav-1 silencing also increased the expression levels of these proteins in senescent fibroblasts, with further increases observed in response to HIFU treatment (Figure 7F,G).

Next, we evaluated whether decreased Cav-1 expression in response to HIFU application is due to the thermal effect of HIFU. We exposed senescent fibroblasts to hyperthermic conditions for 1 min, based on the temperatures measured in pig skin during HIFU application at 0.3, 0.5, and 0.7 J in LINEAR and DOT modes. Although the mRNA expression levels of CAV-1 in senescent fibroblasts were lowest in response to HIFU delivered at 0.5 J in LINEAR mode, CAV-1 expression in senescent fibroblasts increased in response to hyperthermic conditions. A similar pattern was observed for DOT mode, in which CAV-1 expression decreased in response to HIFU application but increased under hyperthermic conditions (Figure S5A). However, the HSP70 mRNA expression in senescent fibroblasts increased in response to HIFU application in both LINEAR and DOT modes and in response to hyperthermic conditions (Figure S5B). The expression level of *P16* mRNA in senescent fibroblasts was the lowest in response to HIFU applied at 0.5 J in LINEAR mode but increased in response to hyperthermic conditions (Figure S5C).

### 3.8. HIFU Induces Collagen and Elastin Fiber Accumulation 

**Figure 8:**
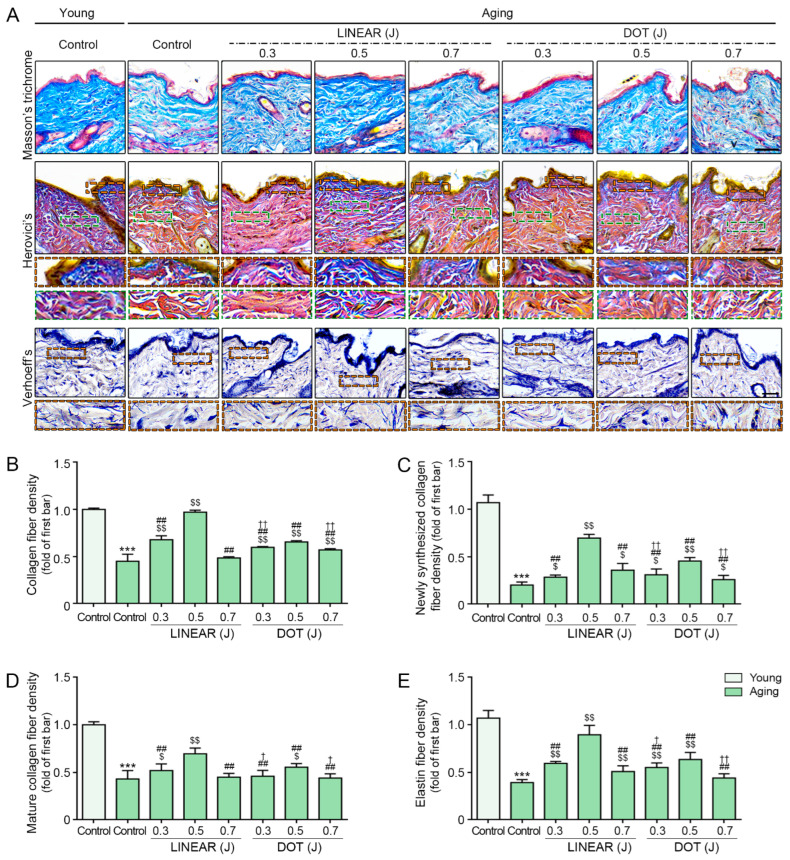
Effect of HIFU application on collagen and elastin fibers. (**A**) Masson’s trichrome to verify collagen fiber, Herovici’s staining to verify newly synthesized and mature collagen fiber, and Verhoeff’s staining to verify elastin fiber in skin from young and aging mice (scale bar for Masson’s trichrome and Herovici’s staining, 50 µm; scale bar for Verhoeff’s staining, 30 µm). The orange dotted box indicates papillary dermis, and the green dotted box indicates reticular dermis. (**B**) Quantification of Masson’s trichrome staining from panel (**A**). (**C**,**D**) Quantification of Herovici’s staining from panel (**A**). (**E**) Quantification of Verhoeff’s staining from panel (**A**). Data were validated with at least three replicates for each experiment (*n* = 3) and are presented as the mean ± standard deviation. HIFU, High-intensity focused ultrasound. ***, *p* < 0.001 for first bar vs. second bar; $, *p* < 0.05, $$, *p* < 0.01 vs. second bar; ##, *p* < 0.01 vs. fourth bar; †, *p* < 0.05, ††, *p* < 0.01 vs. seventh bar.

Our findings indicate that HIFU increases the expression of collagen- and ELN-related proteins. Therefore, we performed histological staining analyses to determine how HIFU affects the levels of collagen and elastic fibers in skin. As expected, Masson’s trichrome staining revealed reduced levels of collagen fibers in the skin of aging mice compared with the skin of young mice (Figure 8A,B). However, collagen levels increased in mice treated with HIFU, with the most prominent increase detected in response to HIFU delivered at 0.5 J in LINEAR mode.

We then used Herovici’s staining to assess newly formed collagen (stained blue) and mature collagen (stained red) in HIFU-treated mice [56,57]. The results showed lower densities of both newly synthesized and mature collagen fibers in the skin of aging mice than in the skin of young mice, but the densities of both increased in HIFU-treated animals, particularly those treated with HIFU at 0.5 J in LINEAR mode (Figure 8A,C,D).

We performed Verhoeff’s staining to evaluate the elastic fiber density in the dermis. We detected a lower density of elastic fibers in the skin of aging mice than in the skin of young mice. Consistent with our earlier observations, elastic fiber densities increased in response to HIFU treatment, with the most prominent effects detected in mice that received HIFU at 0.5 J in LINEAR mode (Figure 8A,E).

The epidermal thickness in the aging mice was lower than in the young mice, and thickness increased in response to HIFU, with the most prominent effect observed for mice treated with HIFU at 0.5 J in LINEAR mode (Figure S6).

## 4. Discussion

We evaluated whether HIFU results in enhanced skin rejuvenation effects via the modulation of Cav-1 activity using an in vitro H_2_O_2_-induced senescent fibroblast model and mouse models of young and aging skin. Because changes in tissue temperatures in response to HIFU can vary depending on the application mode and the delivered energy level, we evaluated changes in Cav-1 expression in response to HIFU applied using both LINEAR and DOT modes at energy levels of 0.3, 0.5, and 0.7 J. Using ex vivo pig skin, we found that tissue temperatures increased with the use of increased HIFU energy outputs. At 0.3 J, no significant differences in tissue temperatures were observed between the DOT and LINEAR modes. However, the tissue temperatures in response to the LINEAR mode at 0.5 and 0.7 J were significantly lower than the temperatures in response to the DOT mode at the same energy output levels.

The DOT mode generates focal points at regular intervals, whereas the LINEAR mode generates uninterrupted linear energy at the target area [51]. The thermal image of ex vivo pig skin showed that LINEAR mode generated a broader thermal area than the DOT mode. 

The thermal effect of HIFU increased HSP70 expression in senescent fibroblasts, with increased HSP70 expression associated with higher HIFU energy levels. At the same energy levels, HSP70 expression was higher in response to DOT mode than in response to the LINEAR mode. The expression trend observed for HSP70 in response to HIFU application was similar to trends observed for temperature changes in ex vivo pig skin, with both increasing in response to increases in HIFU energy output. However, the pattern of Cav-1 expression in response to different HIFU energy output levels did not correlate with the patterns observed for HSP70 expression or temperature. Cav-1 expression in senescent fibroblasts presented with the most prominent decrease in response to HIFU delivered at 0.5 J, with lower Cav-1 expression observed in response to the LINEAR mode than in response to the DOT mode.

The caveolar density in the plasma membrane changes in response to mechanical stress, as caveolae are linked to the cytoskeleton, and mechanical stress can induce the softening and fluidization of the cytoskeleton [61,62]. Ultrasound waves induce particle displacement, resulting in cytoskeletal fluidization [63]. Moreover, increases in temperature above the supra-physiological level can induce increases in membrane fluidity [64].

When forming our hypothesis, we speculated that the thermal effects of HIFU might modulate Cav-1 expression. Contrary to our speculation, our results show that Cav-1 expression in senescent fibroblasts was most prominently suppressed in response to HIFU administered at 0.5 J in LINEAR mode, suggesting that the effects of HIFU on Cav-1 expression are not dependent on thermal effects. The mRNA expression of Cav-1 was lower in response to HIFU application than in response to hyperthermic conditions, similarly to those generated by HIFU application. However, the mRNA expression of HSP70 was increased by both HIFU application and similar hyperthermic conditions. The p16 expression was also the lowest in response to HIFU administered at 0.5 J in LINEAR mode; however, p16 expression in senescent fibroblasts increased in response to hyperthermic conditions. These findings suggest that hyperthermic conditions and the increased temperatures generated in response to HIFU application both increase HSP70 expression, whereas Cav-1 expression may be differentially affected by both temperature and mechanical stimulation, resulting in a different pattern in response to HIFU treatment from that observed for HSP70. Moreover, the use of higher or lower energy output levels than 0.5 J was less effective for reducing Cav-1 expression. We speculate that both proper temperature generation and mechanical stimulation are required to maximize the effects of HIFU application on Cav-1 expression. However, we could not decisively state why Cav-1 expression was most prominently suppressed when HIFU was applied at 0.5 J in LINEAR mode, as we did not specify the mechanism through which HIFU decreases Cav-1 expression in the present study.

In the skin of aging mice, the application of HIFU in LINEAR mode resulted in a greater decrease in Cav-1 expression than the application of HIFU in DOT mode, even at the same energy output level. The LINEAR mode generates a broader energy area than DOT mode. As we applied HIFU using the same frequency (7 MHz) and focal depth (0.5 mm) for both modes, the differences between outcomes for the DOT and LINEAR modes may have been due to differences in the generated energy area. Thus, we speculate that the LINEAR mode induced a larger reduction in Cav-1 expression because it affected a broader tissue area than the DOT mode.

Cav-1 promotes the downregulation of the ERK1/2 signaling pathway in senescent cells [7]. Consistent with this model, we found that HIFU application increased ERK1/2 phosphorylation in senescent fibroblasts, with the most prominent increase observed when HIFU was applied at 0.5 J in LINEAR mode. Moreover, decreased Cav-1 expression in HIFU-treated mice was accompanied by increased ERK1/2 phosphorylation. Similar to our findings with Cav-1, HIFU had the strongest impact on ERK1/2 phosphorylation at the 0.5 J level compared with HIFU delivered at 0.3 or 0.7 J.

Endogenous Cav-1 increases during the G0/G1 phase, but Cav-1 overexpression can induce cell cycle arrest via increased p53 activity [5]. Cav-1-mediated p53 activation results from binding between Cav-1 and MDM2, which prevents MDM2 from binding p53 [22]. Decreased binding between p53 and MDM2 inhibits p53 degradation, leading to increased p53 levels [22]. Cav-1 also sequesters Sirt1 in the caveolar membrane, inhibiting Sirt1 activity and resulting in increased p53 acetylation and premature cellular senescence [23].

In our study, we evaluated whether HIFU application affects the binding of Cav-1 with MDM2 or Sirt1 in senescent fibroblasts and the skin of aging mice. Our data show that both interactions are decreased by HIFU application, with a maximal effect observed for HIFU applied at 0.5 J in LINEAR mode. HIFU also decreases both total p53 and ace-p53 levels in senescent fibroblasts and the skin of aging mice, accompanied by reduced p21 levels and increased levels of proteins involved in S-phase entry, including cyclin D1 and CDK2, with the most prominent changes also observed when HIFU was applied at 0.5 J in LINEAR mode. In addition, HIFU treatment resulted in decreased MMP1 and increased TIMP1 expression, as well as increased synthesis of collagen and ELN.

Aging skin is characterized by gradual thinning and the loss of skin volume due to reductions in ECM component levels, such as ELN and collagen [12]. In humans, collagen levels begin to decrease starting at 30 years of age, mainly in the dermis [12]. Collagen fibers act as a matrix that supports the skin and maintains elasticity [65,66]. The primary collagen types responsible for maintaining skin architecture are types I and III [67]. Therefore, collagen regeneration is essential for skin rejuvenation [68]. We found that HIFU application increases collagen fiber accumulation in the skin of aging mice due to elevated levels of both newly synthesized and mature collagen. Thus, our results show that HIFU application can increase collagen levels in aging skin, with a maximal effect observed when HIFU is applied at 0.5 J in LINEAR mode.

Elastogenesis begins with the generation of tropoelastin monomers, which form the tropoelastin complex by binding with EBPs in ELN-synthesizing cells, such as fibroblasts [69]. The tropoelastin complex is secreted into the extracellular space, where it associates with microfibrils, the scaffolds of ELN fibers [69,70,71]. ELN fibers have a slow turnover rate, with a half-life of almost 70 years [72,73]. However, during aging, ELN levels gradually decrease, resulting in the loss of skin elasticity [74]. Because de novo elastogenesis only occurs until adolescence under natural circumstances and during the wound healing process in adults [75], ELN fibers are not easily replaced [72,76]. Although several treatments have been developed to trigger collagen fiber synthesis, such as collagen injection, procedures designed to increase ELN fiber synthesis have yet to be developed [77]. The current strategy for preventing ELN fiber loss is protection against UV radiation and oxidative stress [77]. In the present study, we found that HIFU application promotes ELN and EBP expression and results in a greater ELN fiber density in the skin of aging mice. These data suggest that HIFU may trigger elastogenesis, indicating that HIFU may be an ideal modality for skin rejuvenation through the induction of both collagen and ELN fiber synthesis. Moreover, our results show that HIFU application increases epidermal thickness.

**Figure 9:**
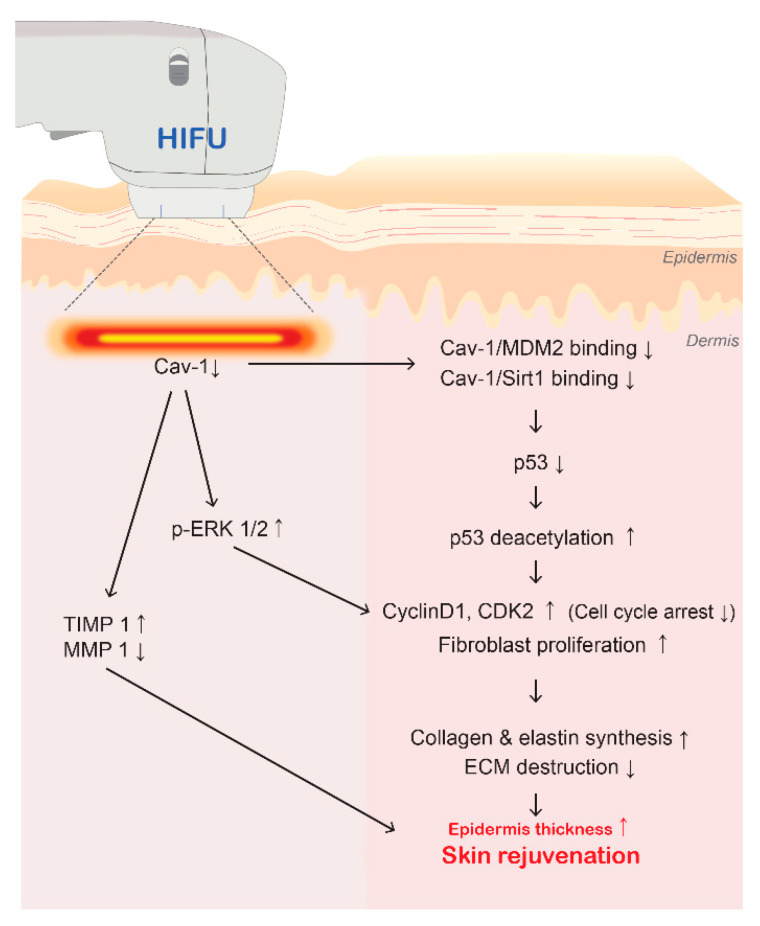
Schematic summary of study results. HIFU application at 0.5 J in LINEAR mode effectively decreases Cav-1 expression (↓) and reduces binding of Cav-1 with both MDM2 and Sirt1 (↓), accompanied by decreased p53 levels and reduced p53 activation (↓), inhibiting cell cycle arrest (cyclin D1 and CDK2; ↓). Also, fibroblast proliferation increased (↑). These changes led to promoted collagen and elastin synthesis (↑) and decreased ECM destruction (↓). Moreover, decreased Cav-1 expression was accompanied by increased p-ERK1/2 (↑), further increasing fibroblast proliferation (↑). HIFU application also increased TIMP1 (↑) and reduced MMP1 levels (↓) in the skin of aging mice. Together, these results suggest that HIFU may be able to increase epidermal thickness (↑) and promote collagen and elastin fiber synthesis (↑) in aging skin, thereby enhancing skin rejuvenation. Cav-1, caveolin 1; CDK2, cyclin-dependent kinase 2; EBP, elastin-binding protein; ERK1/2, extracellular signal-related kinase; p-ERK1/2, phosphorylated-ERK1/2; HIFU, high-intensity focused ultrasound; MDM2, mouse double minute 2 homolog; Sirt1, sirtuin 1; MMP1, matrix metalloproteinase 1; TIMP 1, tissue inhibitor of metalloprotease 1.

We evaluated whether HIFU induced skin rejuvenation via Cav-1 modulation by silencing Cav-1 in senescent fibroblasts. MMP1 expression was decreased by both HIFU application and Cav-1 silencing in senescent fibroblasts. On the contrary, TIMP1 expression was increased by silencing Cav-1 in senescent fibroblasts. Collagen and ELN synthesis were also increased by both HIFU application and Cav-1 silencing in senescent fibroblasts. These findings suggest that HIFU application increases collagen and ELN synthesis via Cav-1 modulation (Figure 9). However, the exact mechanism through which HIFU application modulates Cav-1 activity could not be determined by our present study and should be the focus of future studies.

## 5. Conclusions

In conclusion, we showed that HIFU treatment at 0.5 J in LINEAR mode effectively decreases Cav-1 expression and reduces the binding of Cav-1 with both MDM2 and Sirt1. These changes are accompanied by decreased p53 and p21 levels, and reduced p53 activation, reducing cell cycle arrest and leading to fibroblast proliferation and collagen and ELN synthesis. The observed decrease in Cav-1 expression induced by HIFU was accompanied by increased ERK1/2 phosphorylation, further increasing fibroblast proliferation. MMP1 expression was also reduced by HIFU application to the skin of aging mice. Together, these results suggest that HIFU application may be able to promote collagen and ELN fiber synthesis in aging skin (Figure 9).

## Data Availability

All data are contained within the article.

## Supplementary Materials

The following supporting information can be downloaded at: https://www.mdpi.com/article/10.3390/cells12182275/s1, Figure S1: Schematic illustration of the experimental setup for measuring skin temperature after HIFU treatment; Figure S2: The thermal pattern observed when applying HIFU in LINEAR and DOT modes; Figure S3: Effects of HIFU treatment on p16 and HSP70 expression in senescent fibroblasts and the skin of aging mice; Figure S4: Effects of HIFU application on collagen types I and III levels in the skin of aging mice; Figure S5: Correlating HIFU-induced increases in Cav-1 expression with thermal effects; Figure S6: Effects of HIFU application on epidermal thickness; Table S1: List of antibodies for Western blot, co-immunoprecipitation (Co-IP), enzyme-linked immunosorbent assay (ELISA) and 3,3′-diaminobenzidine (DAB) staining; Table S2: List of primers for qRT-PCR; Table S3: Skin temperature in response to HIFU applied in LINEAR and DOT modes with different energy intensities.
