# Supplementary material for: High-Intensity Focused Ultrasound Increases Collagen and Elastin Fiber Synthesis by Modulating Caveolin-1 in Aging Skin
Source: Cells. 2023 Sep 14;12(18):2275. doi: 10.3390/cells12182275 (PMC10527789; doi:10.3390/cells12182275)
Supplement: Supplementary file 1 [file cells-12-02275-s001.zip › cells-2555057-supplementary.pdf]

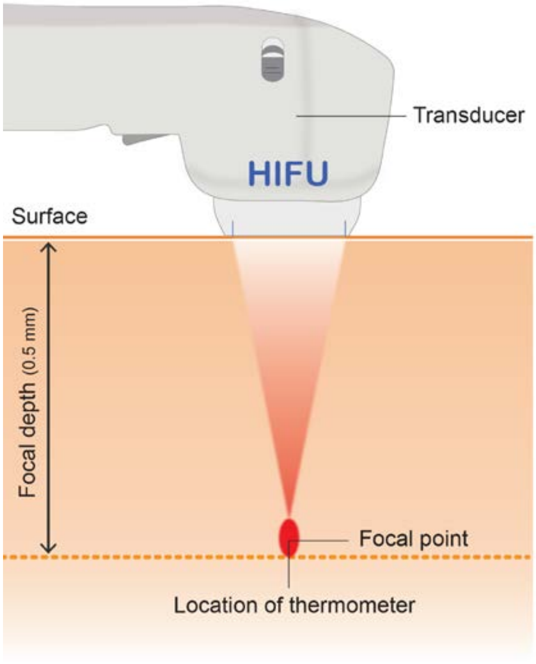


**Figure S1**. Schematic illustration of the experimental setup for measuring skin temperature after HIFU treatment. HIFU, high-intensity focused ultrasound.

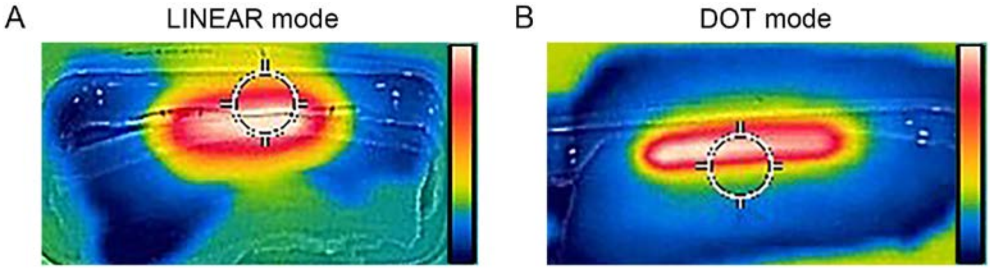


**Figure S2**. The thermal pattern observed when applying HIFU in LINEAR and DOT modes. Thermal images of porcine skin in response to HIFU application at 0.7 J in LINEAR (**A**) or DOT (**B**) mode. HIFU, high-intensity focused ultrasound. HIFU, high-intensity focused ultrasound.

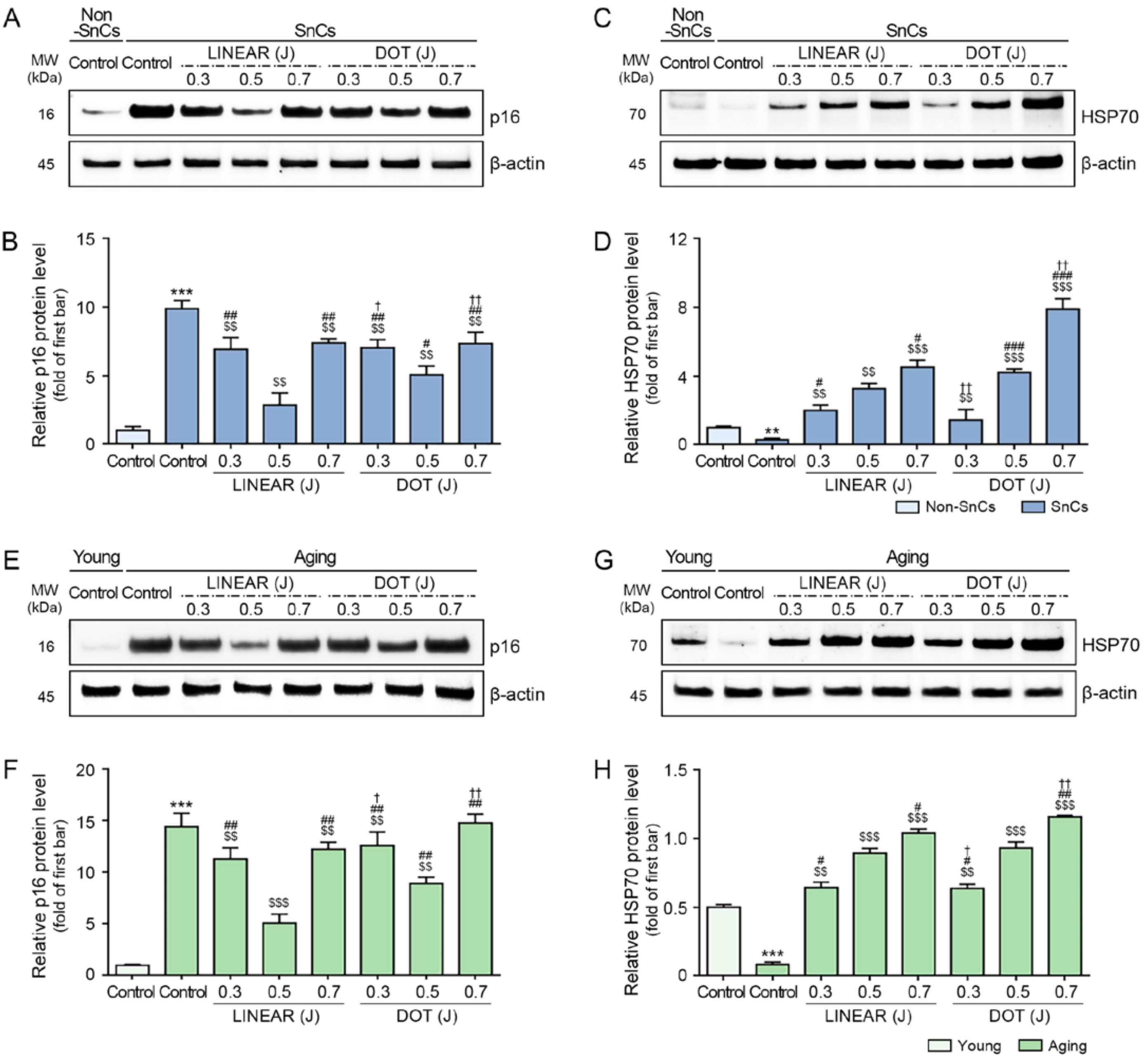


**Figure S3.** Effects of HIFU treatment on p16 and HSP70 expression in senescent fibroblasts and the skin of aging mice. (**A**) p16 protein expression in fibroblasts was evaluated by Western blot. (**B**) Quantification of the data in panel A. p16 expression in SnCs was higher than that in non-SnCs. p16 expression in SnCs treated with HIFU was lower than that in untreated SnCs. The lowest p16 expression level was recorded in response to HIFU delivered at 0.5 J in LINEAR mode. (**C**) HSP70 protein expression in fibroblasts was evaluated by Western blot. (**D**) Quantification of the data in panel C. HSP70 expression in SnCs was lower than that in non-SnCs. HIFU application increased HP70 protein expression, with the highest HSP70 expression level observed in response to HIFU delivered at 0.7 J in DOT mode. (**E**) p16 protein expression in skin tissues was evaluated by Western blot. (**F**) Quantification of the data in panel E. p16 expression in the skin of aging mice was higher than that in the skin of young mice. HIFU application decreased p16 expression in the skin of aging mice compared with untreated skin, and the lowest p16 expression level was observed in response to HIFU applied at 0.5 J in LINEAR mode. (**G**) HSP70 protein expression in skin tissues was evaluated by Western blot. (**H**) Quantification of the data in panel G. HSP70 expression was lower in the skin of aging mice than in the skin of young mice. HSP70 expression in the skin of aging mice was higher in response to HIFU application than in untreated skin, with the highest HSP70 expression level observed in response to HIFU delivered at 0.7 J in DOT mode. Band intensities of single blots were quantified, and expression levels are presented relative to the level indicated by the first bar in the graph after normalization to β-actin levels (loading control). Data are presented

as the mean ± standard deviation. HIFU, high-intensity focused ultrasound; HSP70, heat shock protein 70; MW, molecular weight; Non-SnCs, non-senescent cells; SnCs, senescent cells. **, $p < 0.01$, ***, $p < 0.001$ for first bar vs. second bar; $$, $p < 0.01$, $$$, $p < 0.001$ vs. second bar; #, $p < 0.05$, ##, $p < 0.01$, ###, $p < 0.001$ vs. fourth bar; †, $p < 0.05$, ††, $p < 0.01$ vs. seventh bar.

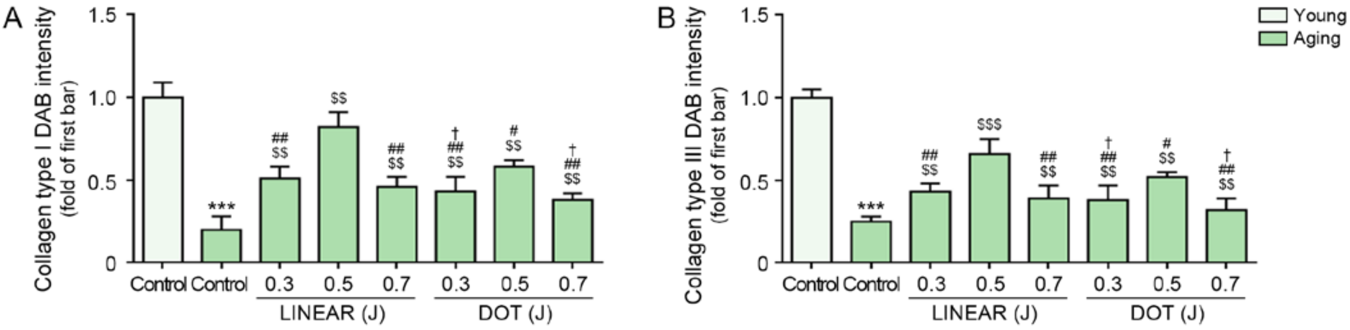


**Figure S4.** Effects of HIFU application on collagen types I and III 3 levels in the skin of aging mice. (**A**,**B**) Quantification graph of collagen types I (**A**) and III (**B**) in Figure 6. The DAB intensities are presented relative to those indicated by the first bar in the graph. Data were validated with at least three replicates for each experiment (n = 3) and presented the mean ± standard deviation. ***, $p < 0.001$ for first bar vs. second bar; $$, $p < 0.01$ vs. second bar; #, $p < 0.05$, ##, $p < 0.01$ vs. fourth bar; †, $p < 0.05$ vs. seventh bar. DAB, 3,3′-diaminobenzidine; HIFU, high-intensity focused ultrasound.

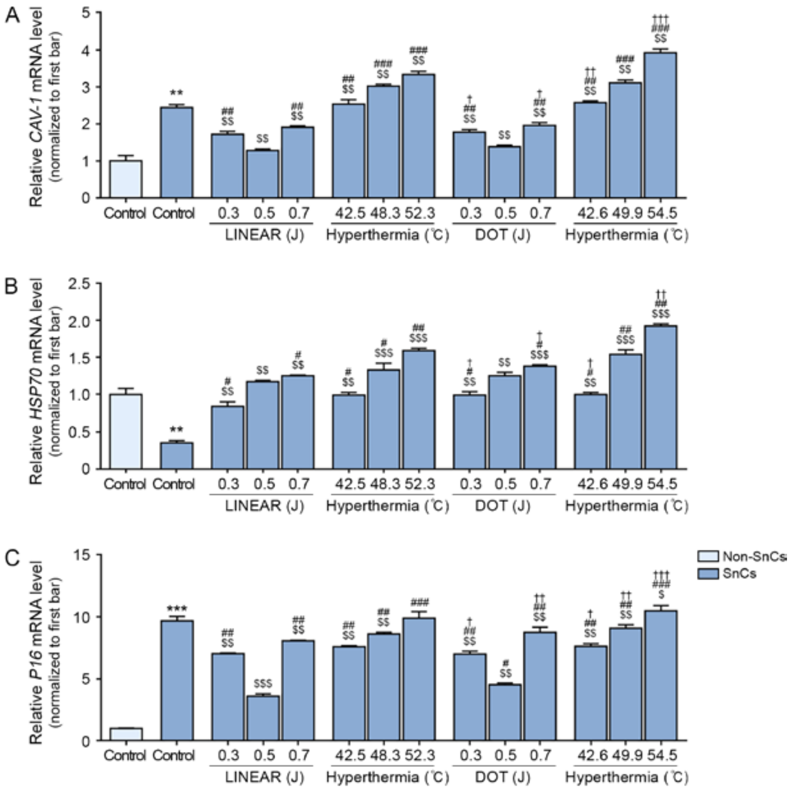


**Figure S5.** Correlating HIFU-induced increases in Cav-1 expression with thermal effects. (**A–C**) The mRNA expression levels of CAV-1 (**A**), HSP70 (**B**), and P16 (**C**) in senescent fibroblasts were measured by quantitative real-time–polymerase chase reaction. The mRNA expression levels of CAV-1, HSP70, and P16 increased in SnCs under hyperthermic conditions. Data were normalized to ACTB levels, and expression levels are reported relative to the level indicated by the first bar in the graph using the comparative CT method. Data are presented as the mean ± standard deviation. CAV-1, caveolin-1; HIFU, high-intensity focused ultrasound; HSP70, heat shock protin 70; Non-SnCs, non-senescent cells; SnCs, senescent cells. **, $p < 0.01$, ***, $p < 0.001$ for first bar vs. second bar; $, $p < 0.05$, $$, $p < 0.01$, $$$, $p < 0.001$ vs. second bar; #, $p < 0.05$, ##, $p < 0.01$, ###, $p < 0.001$ vs. fourth bar; †, $p < 0.05$, ††, $p < 0.01$, †††, $p < 0.01$ vs. seventh bar.

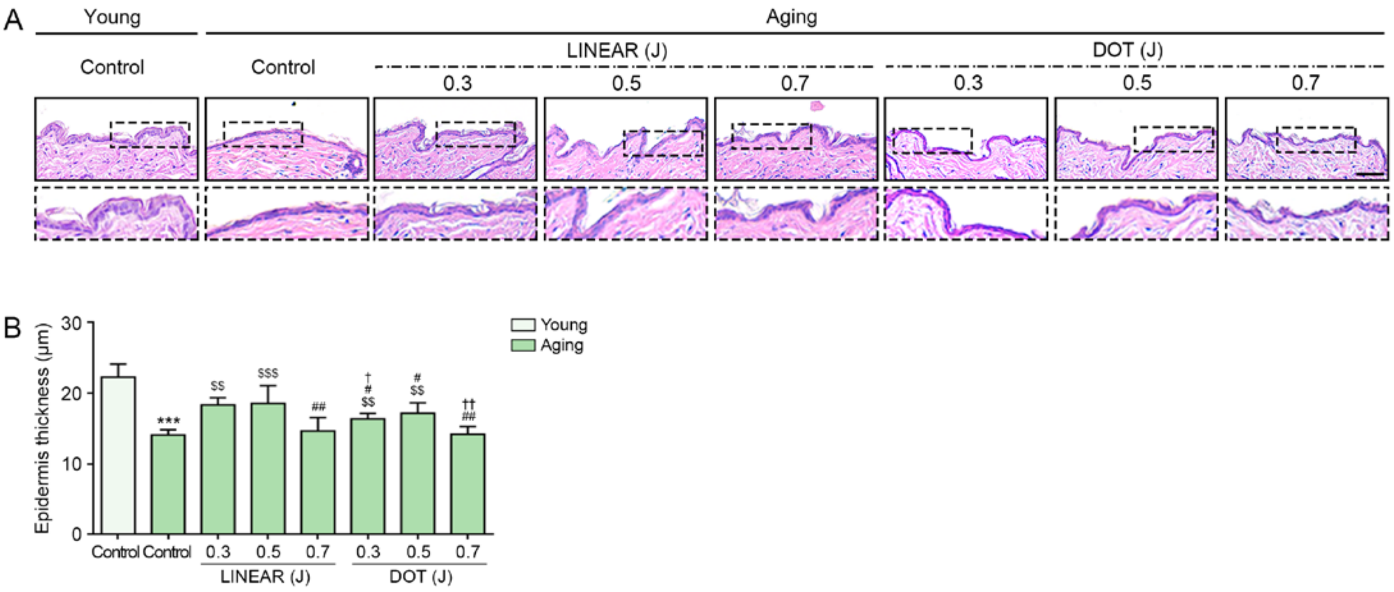


**Figure S6.** Effects of HIFU application on epidermis thickness. (**A**) Hematoxylin and eosin staining of skin from young and aging mice (scale bar, 50 μm). (**B**) Quantification of panel A. Data are presented as the mean ± standard deviation. HIFU, high-intensity focused ultrasound. ***, $p < 0.001$ for first bar vs. second bar; $$, $p < 0.01$, $$$, $p < 0.001$ vs. second bar; #, $p < 0.05$, ##, $p < 0.01$ vs. fourth bar; †, $p < 0.05$, ††, $p < 0.01$ vs. seventh bar.

**Table S1.** List of antibodies for Western blot, co-immunoprecipitation (Co-IP), enzyme-linked immunosorbent assay (ELISA) and 3,3′ -diaminobenzidine (DAB) staining.

| Antibody name | Company | Dilution rate | | | |
|---|---|---|---|---|---|
| | | Western blot | Co-IP | ELISA | DAB |
| Cav-1 | Fine Test | 1:1000 | 1:100 | | |
| p-ERK 1/2 | Cell Signaling Technology | 1:1000 | | | |
| ERK 1/2 | Cell Signaling Technology | 1:1000 | | | |
| MDM2 | Proteintech | 1:1000 | | | |
| Sirt1 | Santa Cruz Biotechnology | 1:1000 | | | |
| p53 | Santa Cruz Biotechnology | 1:1000 | | | |
| ace-p53 | Cell Signaling Technology | 1:1000 | | | |
| p21 | Bioassay Technology Laboratory | 1:1000 | | | |
| CyclinD1 | BioLegend | 1:1000 | | | |
| CDK2 | ABclonal | 1:1000 | | | |
| PCNA | Abcam | 1:500 | | | |
| TIMP1 | St John's Laboratory | | | 1:500 | |

| | | | | |
|---|---|---|---|---|
| MMP1 | Fine Test | | 1:1000 | |
| ELN | Mybiosource | | 1:500 | |
| EBP | LS Bio | | 1:500 | |
| Collagen 1 | Santa Cruz Biotechnology | | | 1:50 |
| Collagen 3 | Bioss | | | 1:100 |
| p16 | Fine Test | 1:1000 | | |
| HSP70 | Cell signaling Technology | 1:1000 | | |
| β-actin | Cell Signaling Technology | 1:1000 | | |

**Table S2**. List of primer for qRT-PCR

| **Gene** | | **Primers** |
|---|---|---|
| *CAV-1* | Forward | 5'-TAC TTC GCC ATT CTC TCT TTC C-3′ |
| | Reverse | 5'-AGA TGG AAT AGA CAC GGC TGA T-3′ |
| *HSP70* | Forward | 5'-TGA GGG TAA GAT GAT CAT GCA G-3′ |
| | Reverse | 5'-ACA TAT TCC TCC ACT GCG TTC T-3′ |
| *P16* | Forward | 5'-ACC AGA GGC AGT AAC CAT GC-3′ |
| | Reverse | 5'-TGC TTC TAC AAA CCC ACA AAT G-3′ |
| *ACTB* | Forward | 5′-GGG ACC TGA CTG ACT ACC TCA T-3′ |
| | Reverse | 5′-CCT TAA TGT CAC GCA CGA TTT-3′ |

**Table S3**. Skin temperature in response HIFU applied in LINEAR and DOT mode with different energy intensities.

| **HIFU** | | **Temperature** |
|---|---|---|
| LINEAR | 0.3 J | 42.48 ± 0.19°C |
| | 0.5 J | 48.32 ± 0.23°C |
| | 0.7 J | 52.30 ± 0.37°C |
| DOT | 0.3 J | 42.64 ± 0.34°C |
| | 0.5 J | 49.94 ± 0.47°C |
| | 0.7 J | 54.52 ± 0.36°C |
